# Solvation Free Energies of Drug-like Molecules via Fast Growth in an Explicit Solvent: Assessment of the AM1-BCC, RESP/HF/6–31G*, RESP-QM/MM, and ABCG2 Fixed-Charge Approaches

**DOI:** 10.1021/acs.jctc.5c00749

**Published:** 2025-08-11

**Authors:** Matteo Orlandi, Yiqi Geng, Marina Macchiagodena, Marco Pagliai, Piero Procacci

**Affiliations:** † Dipartimento di Chimica “Ugo Schiff”, 9300 Università degli Studi di Firenze, Via della Lastruccia 3, 50019 Sesto Fiorentino, Italy; ‡ Dipartimento di Neuroscienze, Psicologia, Area del Farmaco e Salute del Bambino, 9300 Università degli Studi di Firenze, Via Ugo Schiff 6, 50019 Sesto Fiorentino, Italy

## Abstract

We assess the performance of the nonequilibrium alchemical fast-growth method in calculating water and 1-octanol solvation free energies, comparing the recently proposed ABCG2 model with other empirical and quantum mechanics (QM)-based approaches for modeling electrostatic interactions in condensed phases using fixed atomic charges. The fixed-charge protocols are tested on the challenging set of drug-like polyfunctional molecules previously used by Vassetti et al., *J. Chem. Theory Comput.* **2019**, *15*, 1983–1995, broadly spanning the chemical space and often exhibiting complex conformational landscapes. We find that the cost-effective empirical ABCG2 protocol consistently outperforms the AM1/BCC precursor model and the widely used HF/6–31G* *ab initio* charge derivation method, achieving solvation free energy accuracy comparable to an expensive QM/MM-based methodology for atomic fixed charge determination. For water-octanol transfer free energies, ABCG2 benefits from systematic error cancellation, yielding remarkable agreement with experimental data, exhibiting excellent Pearson and Kendall rank coefficients and a mean unsigned error below 1 kcal/mol, matching the performance of the costly QM/MM approach. These results suggest that the ABCG2 protocol holds great promise for the high-throughput in silico prediction of ligand-protein binding free energies in drug discovery projects.

## Graphical abstract

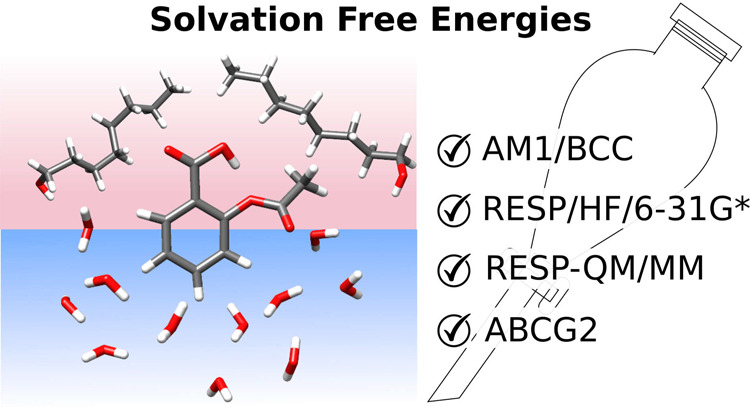

## Introduction

In the past decade, computational approaches in drug design have spurred enormous interest, boosted by the development of advanced computational techniques, the widespread accessibility to massively parallel facilities, and the flood of data on ligand-protein properties.^1^ In this context, a reliable and efficient computational tool for the prediction of solvation free energies can afford the in silico design of potential drugs with suitable aqueous solubility and membrane permeability.^2^ Prediction of solvation free energies is not only important *per se*, but is strictly related to the identification of potential inhibitors for a given protein target, considering the fact that the binding free energy in drug-receptor systems can be rationalized in terms of the difference between the solvation free energy of the drug in bulk solvent and the environment delimiting the binding site of the receptor.^3–5^

Today, properties such as hydration free energies or octanol–water partition coefficients are effectively predicted using a variety of techniques, ranging from cost-effective knowledge-based empirical approaches such as Xlog3 or ALOGP,^6–8^ to ab initio (QM) calculations mixed with trained parametric models for the polarization continuum, as in the COSMO^9^ or SMD^10^ implicit solvation models, and computationally demanding atomistic molecular dynamics (MD) simulations with explicit solvent in combination with alchemical approaches.^11–16^

These various computational methodologies for solvation free energies are periodically tested in *blind* international challenges such as the Statistical Assessment of the Modeling of Proteins and Ligands (SAMPL), a well-established international initiative for advancing the computational techniques in drug design.^17–21^ Most of the MD-based applications in SAMPL challenges make use of empirical force fields (FF) where the electrostatic interactions are modeled using fixed atomic charges, evaluated with protocols that take into account the polarization effects of the environment in a mean-field spirit. MD approaches based on truly polarizable FF’s such as AMEOBA^21–24^ add a considerable extra cost to the simulation, preventing their widespread use, for the time being, as a cost-effective screening tool in high-throughput drug design projects.

In the most recent SAMPL challenges, physical QM-based or molecular mechanics (MM) methods with the assumption of *implicit* solvent are consistently found among the top-performing approaches for the calculation of solvation free energies or Log*P* coefficients,^25–27^ in most cases exceeding the prediction power of the more expensive MD-based alchemical methodologies with *explicit* solvent. However, when the former are applied to host–guest systems in the SAMPL challenges, they turn out to be largely outperformed by the latter.^20,28,29^ This is most likely due to the fact that methods based on implicit solvents are by design unable to take into account phenomena that are crucial in affecting the free energy of the complex, such as microsolvation effects and solute conformational response to a heterogeneous environment.^18,26,29,30^

The vast majority of MD approaches in host–guest SAMPL challenges were implemented using a fixed atomic charge parametrization, like the AM1/BCC^31^ protocol or using overpolarized atomic charges computed in vacuo at the HF/6–31G* level of theory to be used in aqueous solvent.^32^ It has been suggested that the success of the MD-based approach with fixed atomic charges in host–guest binding and in Log*P* calculations is due in part to an error compensation whereby these electrostatic models are, in most cases, excessively polarized in both the host–guest (or 1-octanol) and in the pure solvent environments.^31–33^

In the latest Log*P* SAMPL9 challenge,^27^ among the physical methods, a simple MM/GBSA approach based on the new fixed charge ABCG2 protocol^34,35^ resulted as the most accurate one, outperforming the MD-based methodologies. ABCG2 is the successor of the popular AM1/BCC model for fixed charges. Nonetheless, the very same MM/GBSA approach with ABCG2 atomic charges, when applied to the host–guest SAMPL9 challenge featuring ammonium cationic ligands of the carboxy-pillar[6]­arene macrocyclic host,^36^ was outcompeted by several MD-based approaches. Remarkably, some of these MD submissions were performed using the precursor AM1/BCC model since ABCG2 was not yet released to the public then, showing that their success was due to the methodology rather than to FF. In fact, unlike in MM/GBSA, MD-based approaches with explicit solvent take into account the interplay between conformational dynamics and local microsolvation phenomena related to, e.g., competition between solute–solvent or solute–solute H-bonds.

In the article by Vassetti et al.,^33^ where several force fields were compared using MD performed in explicit solvent, it was shown that the main discrepancies observed in the solvation free energy predictions for a series of polyfunctional drug-like molecules were caused by the electrostatic contribution, i.e. to the fixed-charge parametrization. In the same study, it was shown that deviations from the experimental data tend to be significantly attenuated when the octanol–water transfer free energies are computed.

In the latest AmberTools release,^37^ the AMBER team finally made available to the public the ABCG2 model (an abbreviation of AM1-BCC-GAFF2),^35^ basically an update of the old bond charge corrections (BCC) in the AM1/BCC forerunner model. Such an update involves heavily the optimization of the atomic fixed charges and marginally the GAFF2 reparameterization for Lennard-Jones and bonded interactions.

It is, therefore, of interest to compare the accuracy of the new ABCG2 model for the solvation free energy prediction to its forerunner AM1/BCC and to other well-established QM-based techniques for deriving the atomic fixed charges, using alchemical MD approaches on the same batch of molecules employed by Vassetti et al.,^33^ supplemented with some heterocyclic compounds, a chemical type for which GAFF2/AM1-BCC is known to perform poorly.^21,38^ As experience gained from SAMPL challenges have shown, a good performance of MD-based approaches with explicit solvent on the transfer free energies between water and hydrophobic solvents often implies a good predictive power in host–guest or ligand-protein systems.^17–21,25–27^

In the present study, we show that, while the agreement with the experimental individual solvation energies in water and in 1-octanol is not particularly exciting for the selected batch of polyfunctional molecules, regardless of the fixed-charge approach we use, when the Log*P* (i.e., the transfer free energy) is tested, ABCG2 delivers stunningly accurate predictions with a mean unsigned error of only 0.9 kcal/mol and a Pearson correlation coefficient of 0.97, outperforming the old parametrization AM1/BCC, and providing an accuracy comparable to that obtained using fixed charges calculated at the QM/MM level. These results, besides demonstrating that in computing the solute transfer solvation free energies between aqueous and hydrophobic environment, error compensation plays indeed a crucial role as shown by Vassetti et al.,^33^ authorizes great expectations for the usage of the ABCG2 protocol in the prediction of host–guest or ligand-protein binding free energy.

The paper is organized as follows. In section “Methods” we provide technical data on the FF used in our MD simulations, describing in some detail the nonequilibrium fast-growth approach to compute the solvation energies. In section “Results”, we present the results obtained using the atomic fixed charges computed with AM1-BCC, ABCG2, HF/6–31G*, and the QM/MM protocols. A critical assessment of our results and of the extensive tests recently published by the ABCG2 developers^35^ is provided in the “Discussion” section. Conclusive remarks and perspective of ABCG2 applications in high-throughput drug design projects are discussed in the “Conclusions” section.

## Methods

In Figure 1, we report the structure of the molecules that have been considered in this study.

**Figure 1:**
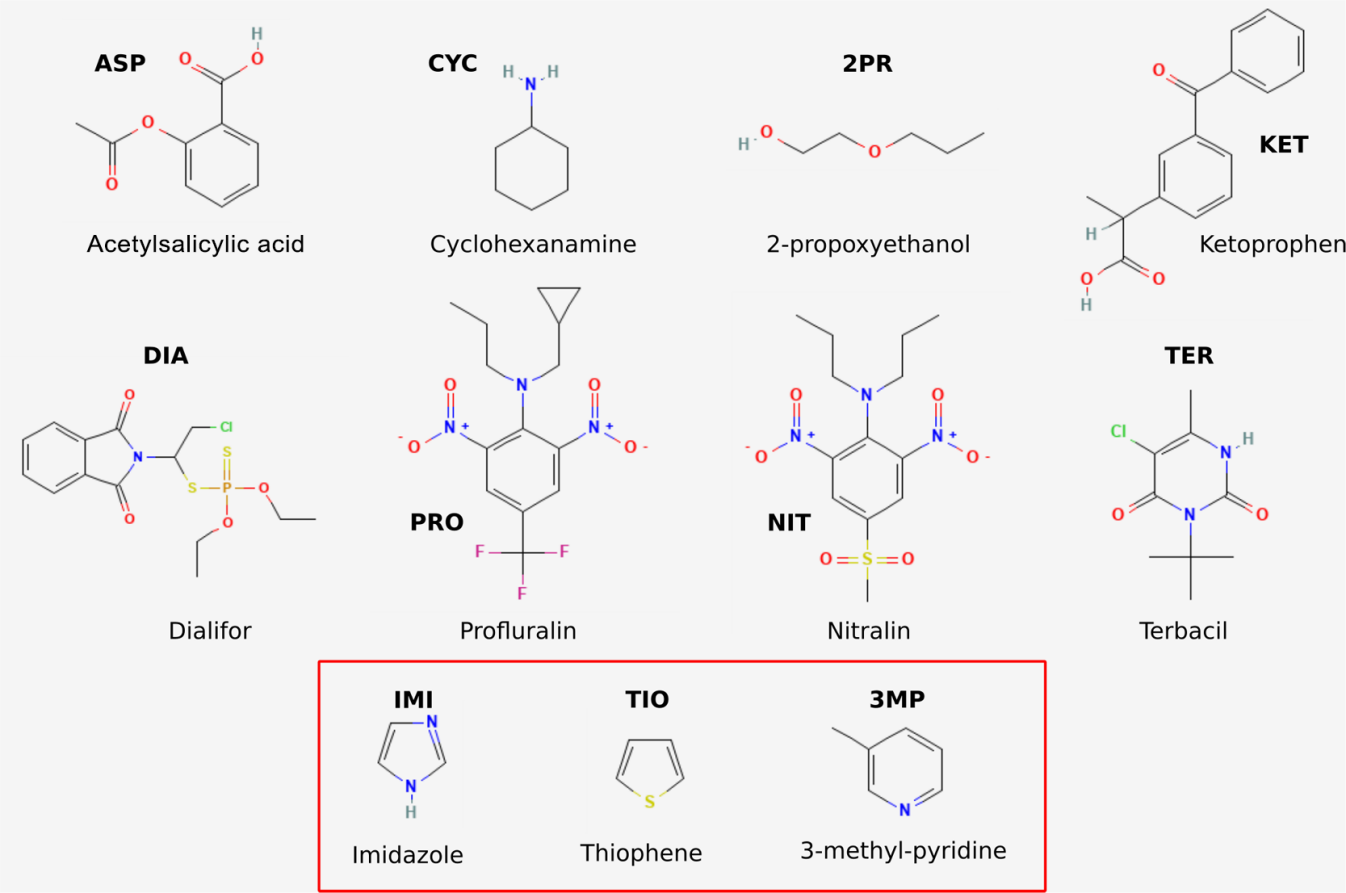
Selected set of molecules.

The three letters in bold will be used throughout the paper when referring to these molecules. The heterocyclic compounds in the red box (IMI, TIO, 3MP) were not included in the study by Vassetti et al.^33^ Except for CYC, IMI, TIO, and 3MP, the compounds are polyfunctional, with a number of heavy atoms ranging from 7 (2PR) to 28 (PRO) and with a total of rotatable bonds greater than five for most molecules. As already stated,^33,35^ this batch of compounds, while limited, constitutes a significant challenge for solvation free energy calculations. In this regard, in the recent assessment of ABCG2 and AM1/BCC parametrization for solvation free energies of hundreds of small molecules,^35^ while the agreement with experimental data was, in general, very satisfactory for most of the compounds, outliers were systematically found for high molecular weight, polyfunctional, flexible molecules. The three heterocyclic compounds IMI, TIO, and 3MP were introduced as GAFF2-based fixed-charge models are known to perform poorly in the solvation free energy and hydrogen-bonding properties of compounds with a lone pair on N and S atoms.^35,38–41^

### QM/MM Molecular Dynamics Simulations

For every molecule shown in Figure 1, an NVT QM/MM MD simulation in water was carried out using the CP2K code interfaced with the GROMACS package^42,43^ for 20 ps under standard conditions. The starting configuration was taken from a classical NPT simulation of the solute in 512 water molecules at *T* = 300 K and *p* = 1 atm, using the GAFF2 model for the solute and the SPC/E model for water.^33^

In the QM/MM simulations, the solute was treated through quantum mechanics, whereas the surrounding water was described classically through the SPC/E model. The QM atoms were described at the DFT level of theory, with the BLYP exchange and correlation functional combined with the DZVP-MOLOPT basis set.^44^ The core electrons of each atom were modeled through Goedecker-Teter-Hutter (GTH) pseudopotentials^45–47^ while the electron density was described with a plane-wave expansion of 450 Ry. Grimme D3 dispersion corrections^48^ were also included, with a cutoff set to 16 Å. The CP2K/GROMACS interface automatically sets the QM box dimensions. No classical charge redistribution was attempted since the net charge of each solute in Figure 1 is zero. Moreover, the interface relies on the so-called Gaussian Expansion of the Electrostatic Potential (GEEP) approach^49^ that smears the classic atomic charges at the QM/MM interface, preventing the electronic spill-out problem from the QM region. The GEEP electrostatic embedding^49^ was enforced using 12 Gaussian functions for the expansion of the MM potential. The equations of motion were integrated with a time-step of 0.5 fs. The temperature was maintained at 300 K using the velocity rescaling algorithm (Bussi-Donadio-Parrinello)^50^ with an inverse friction constant of 0.2 ps. Long-range electrostatics were treated using the Smooth Particle Mesh Ewald method.^51^ The cutoff radius for nonbonded interactions was set to 10 Å.

During the QM/MM simulation, 10 solute configurations were collected by sampling at regular intervals of 2 ps. Fixed atomic charges were later computed, for each of the four protocol AM1/BCC, ABCG2, RESP-QM/MM, RESP/HF/6–31G* (see below for details on the protocols), by averaging over these configurations.

### Fixed Atomic Charges Parameterization of the Solute Molecules

Solvation free energies were computed for all molecules in Figure 1 using the same set of bonded and Lennard-Jones nonbonded parameters (based on GAFF2) but with four different sets of atomic charges, calculated according to the AM1/BCC, RESP/HF/6–31G*, RESP-QM/MM, ABCG2 schemes. AM1/BCC and its improved version, ABCG2, are based on the calculation of the Mulliken charges for the optimized molecular configuration according to the semiempirical Austin Model 1 (AM1),^52^ corrected by so-called bond charge corrections (BCC) that are fitted to reproduce the RESP (Restrained Electrostatic potential)^53^ charges, computed with a full QM approach at the HF/6–31G* level of theory in vacuo. The *in vacuo* RESP/HF/6–31G* charges, despite being in principle unsuited to be used in a strongly polarizing environment, gained enormous popularity for the calculation of properties such as solvation or binding free energies since they are notoriously overpolarized on average by 10–15%,^31,32,54^ hence accounting, by error compensation, for the polarization effect of the solvent in a mean field spirit.

To take into account the modulation of electron density due to internal vibration/rotation,^55^ rather than using the single optimized configuration (i.e., the solute structure at 0 K), the atomic charges in the four sets were averaged over the ten conformations sampled in the QM/MM simulations according to the following protocols:

- ABCG2 atomic charges were calculated using the antechamber command provided by the AmberTools distribution.^56,57^

- The AM1/BCC atomic charges were computed using the primadorac.bash^58^ command-line application provided by the ORAC distribution.^59^
- The RESP/HF/6–31G* atomic charges were obtained in vacuo at the Hartree–Fock level of theory, using the CP2K program.^43^ All-electron calculations were carried out with the Gaussian Augmented Plane Wave (GAPW) method.^60^
- RESP-QM/MM atomic charges were computed at the same level of theory (BLYP-D3) as the one chosen to carry out the preliminary QM/MM MD simulations. The 10 frames extracted from each ligand QM/MM MD simulations were employed to perform single-point electron density optimizations. Therefore, polarization on each solvated ligand (QM) was taken into account through the presence of water molecules (MM employing the SPC/E model), explicitly included in the 10 chosen conformations. Then, polarized electron densities were employed for the standard RESP fitting on the structure of the solutes using CP2K. All the other CP2K settings, regarding the level of theory and calculation set up were left unchanged from the QM/MM MD simulations previously reported. Such a QM/MM RESP protocol is designed to capture explicitly the polarization of the ligands induced by the surrounding water molecules, especially on “hot spots” like heteroatoms that can be involved in hydrogen bond networks.

The four sets of charges belonging to the 11 solutes, depicted in Figure 1, along with the GAFF2 atomic type assignment, are reported in the Supporting Information (SI) in Tables S1–S11. Standard deviations, evaluated on the ten conformations sampled in the QM/MM simulation, are not reported since they are small for all protocols, even for large negative or positive atomic charges in polyfunctional and flexible compounds such as DIA, NIT, or PRO $\left(\frac{\delta q}{\left|q\right|}≃0.03-0.06\right)$ .

In Figure 2, we show the average atomic charge per GAFF2 atomic type computed with the four fixed-charge protocols above-described. While carbon and hydrogen atomic charges are quite similar among the protocols irrespective of the atomic type, major differences can be seen on the right of the plot for atomic types involving nitrogen, phosphorus, and sulfur atoms. For example, the charge of the type n3 (sp^3^ nitrogen in primary amines) is close to −1 *e* for RESP/HF/6–31G*, RESP-QM/MM and ABCG2, and only −0.5 *e* for AM1/BCC. The negative charge on amide nitrogen n in ABCG2 increases by ≃40% compared to the other protocols.

**Figure 2:**
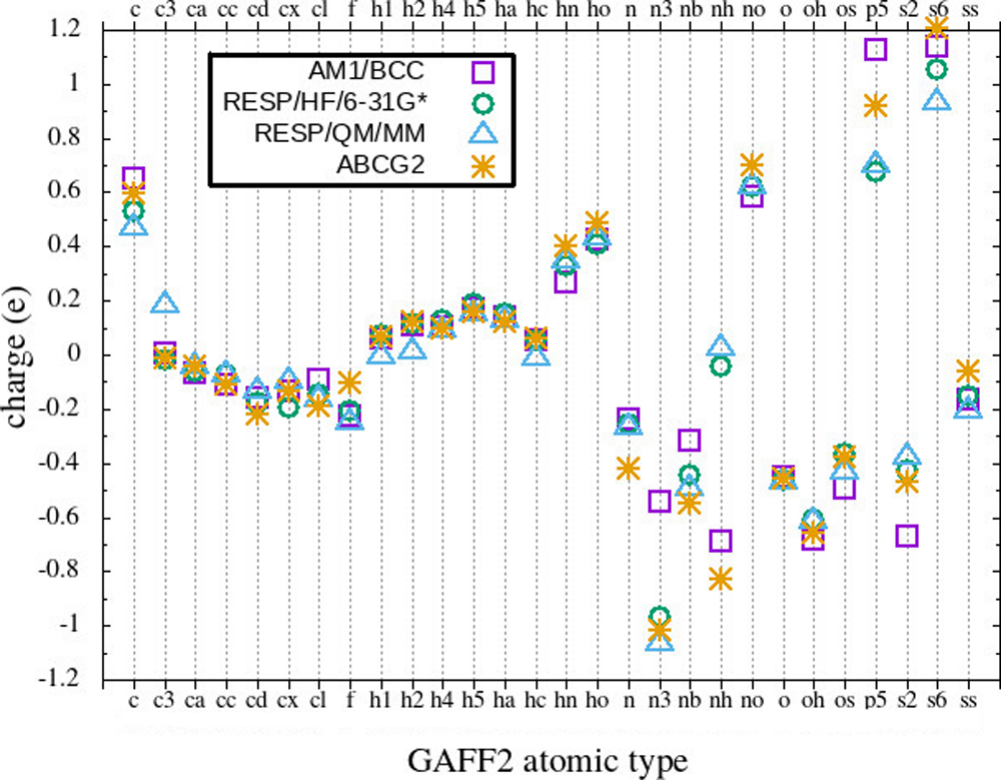
Average charges per GAFF2 atomic types computed with the four fixed-charge protocols. Violet squares: AM1/BCC; green spheres: RESP/HF/6–31G*; cyan triangles: RESP-QM/MM; orange stars: ABCG2.

### Solvent Parameterization

The water solvent was modeled using the SPC/E parametrization.^61^ This model accurately reproduces the density and the dielectric constant of liquid water^33,62^ and was found to perform slightly better for solvation free energy prediction when compared to the TIP3P^63^ and OPC3 ^64^ water models.

The 1-octanol solvent parametrization was obtained by way of the antechamber tool with the ABCG2 model, yielding a density and a dielectric constant of 0.816 g/mL and 6.24, respectively, for the pure liquid at standard conditions, to be compared to the corresponding experimental values of ρ = 0.827 g/mL and ϵ = 9.80.

### General MD Setup

All 11 solute molecules were considered in their neutral form. Solvation free energies were evaluated by dissolving the selected solutes in about 500 water molecules or 340 octanol molecules in a cubic MD box. All simulations were conducted in the NPT isothermal–isobaric ensemble, yielding a mean side-length around 30 and 45 Å in water and 1-octanol, respectively. The external pressure was set to 1 atm using a Parrinello–Rahman Lagrangian^65^ while the temperature was held constant at 300 K using the Bussi-Donadio-Parrinello thermostat.^50^ The equations of motion were integrated with a time-step of 1 fs. Long-range electrostatics were treated using the Smooth Particle Mesh Ewald method,.^51^ The cutoff radius for nonbonded interactions was set to 10 Å except for large-size solutes for which the cutoff was extended up to 14 Å to allow the calculation of the transfer free energy from the solution to the gas-phase using the alchemical methods.^66^ All calculations were performed using the GROMACS program, version 2022.3.^42^

### Fast Growth Method (FGM) for the Solvation Free Energy

The theoretical background of the FGM alchemical methodology has been thoroughly described elsewhere.^27,67,68^ Here we briefly summarize the salient technical aspects of FGM as long as solvation free energy calculations are concerned.

In the FGM method, we gradually recouple in a swarm of nonequilibrium (NE) alchemical trajectories, according to a common time schedule, an initially decoupled (ghost) solute in an equilibrated solvent. The initial configurations of the decoupled solute are prepared by using Hamiltonian Replica Exchange (HREM) sampling^69–71^ on a single solute molecule in the gas-phase with the full intramolecular potential. The HREM parameters (number of replicas and potential energy scaling protocol) are common to all solutes and are identical to those used in ref 27. Gas-phase HREM simulations were performed using the ORAC program,^59^ and lasted 8 ns collecting 200 solute configurations by sampling at regular intervals.

The starting states for the alchemical recoupling trajectories were prepared by combining the HREM-generated gas-phase 200 configurations with a snapshot taken from a simulation of the pure solvent under standard conditions. Each of the corresponding 200 NE alchemical trajectories lasted 2.0 ns using the GROMACS option intramol = no, whereby only the solute–solvent nonbonded interactions are switched on during the alchemical process, leaving the intramolecular force field invariant.

The alchemical protocol dictates that Lennard-Jones interactions are linearly switched on first in 1.2 ns using a soft-core regularization,^72^ followed by the recharging process in the final 0.8 ns. The FGM calculation of any given solute in water or 1-octanol was carried out in single parallel job on the Leonardo “Booster” partition at CINECA,^73^ engaging 50 nodes for a total of 200 GPUs each of which was running a NE recoupling trajectory. The solvation free energies were computed from the histograms of the alchemical recoupling work using the Jarzynski^74^ or Gaussian formula^75^ depending on the character of the work distribution.

The character of the work distribution is examined using standard normality tests, like the Anderson-Darling test^76–78^ (ADT) and the solvation free energy is recovered, in case of ADT fulfillment, by exploiting the Crooks theorem^79^ for normal distribution,^80,81^ i.e.:

$$\Delta G=\left\langle W\right\rangle -\frac{\beta \sigma^{2}}{2}$$ 1

where β = 1/*k*_B_ *T* and ⟨*W*⟩, σ^2^ are the mean and variance of the work distribution *P*(*W*). In case of ADT failure, as done in ref 28, we first ascertain that contribution to the work for switching on the solute–solvent Lennard-Jones (*W*_*lj*_) and that for the recharging process (*W*_*qq*_) are two independent random variables with near-zero correlation; then, by combining each of the 200 *W*_*lj*_ with each of the 200 *W*_*qq*_ values producing 40,000 work values, we compute the solvation free energy on the convolution 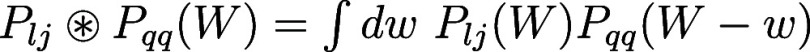 using the Jarzynski identity i.e.:

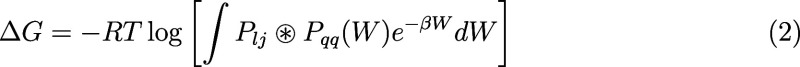

2



## Results

### Work Distribution for Solute Fast-Growth

The work distribution for the fast-growth of the solute in water and 1-octanol was assumed to be normal if the Anderson-Darling test (ADT) was below 0.34 (i.e., *p*-value > 0.5 of the null hypothesis),^82^ computing in that case the solvation free energy using eq 1 with mean and variance determined on the *N* = 200 NE trajectories. In case of 0.34 ≤ ADT ≤ 0.754, the Gaussian estimate, eq 1, was again used if the corresponding Jarzynski estimate $\Delta G=-RT\log\left(\frac{1}{N}\sum_{i}^{N}e^{-\beta W_{i}}\right)$ differs by less than 0.2 kcal/mol. We found only three cases where we used the estimates given in eq 2, hence performing the convolution of Lennard-Jones and recharging work distributions, namely 2PR in water, CYC and TER in 1-octanol for the ABCG2 model.

As an example, in Figure 3 we show the work distributions obtained for 2PR in water (left) and on the right, the correlation plot between Lennard-Jones and recharging work. The Pearson correlation coefficient reported, ρ of −0.03, and Kendall rank coefficient, τ of −0.02, indicate basically zero correlation between the two independent random variables. As a consequence, the resulting well resolved convolution 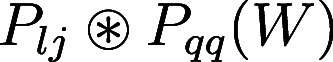 (computed on *N* ^2^ work values) nicely fits the coarse-grained distribution *P*(*W*) of the total work. The Jarzynski estimates eq 2, indicated by the green arrow, occurs on the left tail of the convolution where 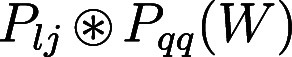 is well sampled, thus significantly reducing the inherent bias of the exponential average.^83^

**Figure 3:**
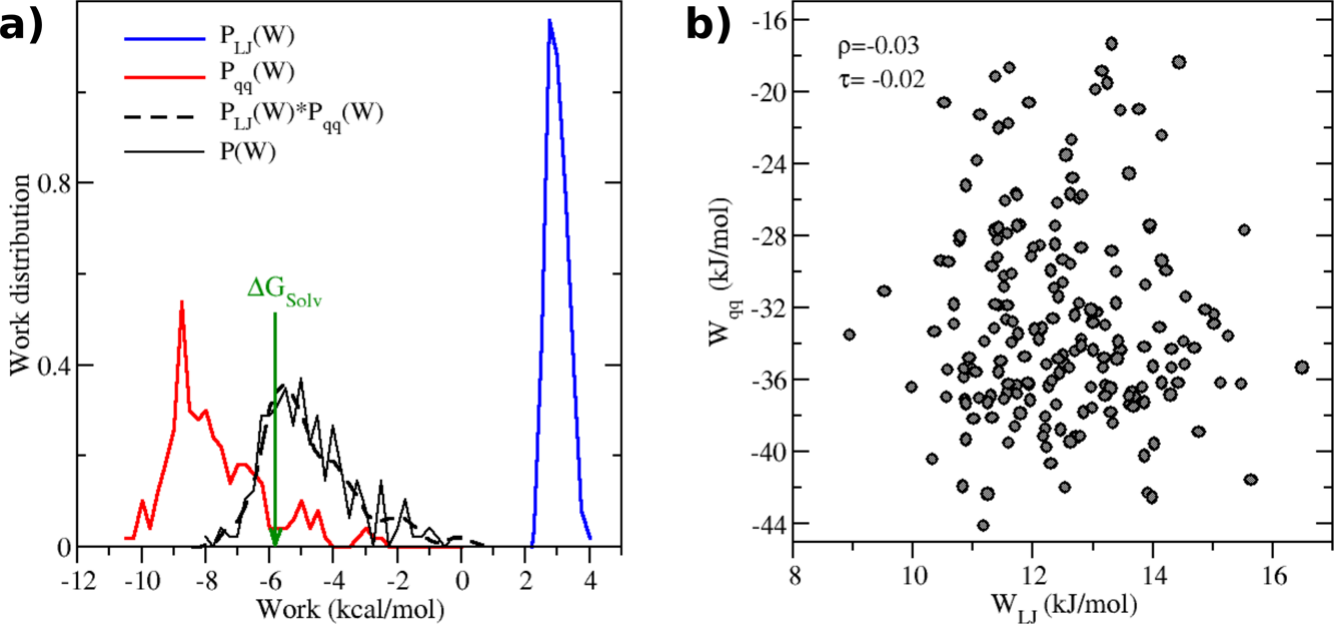
(a) Lennard-Jones (blue), recharging (red), total (solid black), and convolution work distributions for 2PR in water solvent using the ABCG2 model. The green arrow indicates the solvation free energy estimate according to eq 2. (b) Correlation plot between Lennard-Jones and the recharging work. ρ and τ indicates the Pearson and the rank Kendall coefficients, respectively.

### Hydration Free Energy and 1-Octanol Solvation Free Energy Using AM1/BCC, HF/6–31G*, QM/MM, and ABCG2 Protocols

In Tables 1 and 2, we report the computed hydration free energies (Δ*G*_w_) and the solvation free energies in 1-octanol (Δ*G*_0_), respectively. The experimental values for the hydration energies are taken from refs 84 and 85. In case of the Gaussian estimate, eq 1, the 95% confidence intervals are computed using the formula^86^ $\delta \Delta G_{\mathrm{solv}}=z_{\alpha /2}\left|\frac{\sigma_{b/u}}{n^{1/2}}+\frac{1}{2}\beta {\left(\frac{2}{n}\right)}^{1/2}\sigma^{2}\right|$ where 1 – α = 0.95, *z* = 1.96 is the corresponding *z*-score, *n* the number of alchemical processes, σ is the standard deviation, and β = 1/*k*_B_ *T*. When the estimate eq 2 for non-normal distributions is used, the 95% confidence interval is computed from the sum in quadrature of the errors in the Jarzynski estimate of the Lennard-Jones and on the recharging work distribution, evaluated by bootstrap with resampling. For the AM1/BCC model, agreement with experimental Δ*G*_w_ (see Table 1) is unsatisfactory, with the heterocyclic compounds 3MP and IMI (not included in ref 33) severely underestimating the Δ*G*_w_, and with a significant overestimation of the Δ*G*_w_ for the compounds DIA, NIT, and TER. When calculations are performed using directly the RESP/HF/61–31G* charges, i.e., the charges that the AM1/BCC model was trained to emulate,^31^ the overall agreement for the three polyfunctional DIA, NIT, and TER decisively improves. When the RESP-QM/MM charges are used, the agreement further improves, but again we observe a significant overestimation of the Δ*G*_w_ for NIT, DIA and PRO. ABCG2, finally, performs slightly better than the costly RESP-QM/MM approach. It is worth noticing the remarkable improvement in going from the AM1/BCC to the ABCG2 model for the hydration free energies of the heterocyclic IMI, 2MP, and cyclohexanamine CYC. Such effect is due to the bond charge corrections on the nb atom types in 3MP and IMI (see Figure 2 and Tables S9 and S11 of the Supporting Information) that in ABCG2 significantly increases the excess negative charge on the nitrogen. The same applies for the bond charge correction on the n3 atom type in CYC, where the excess charge on the nitrogen and the corresponding positive charges on the hn hydrogen atoms almost double (see Figure 2 and Table S2 of the Supporting Information) in going from AM1/BCC to ABCG2.

**Table 1:** Experimental and Calculated Hydration Free Energies (kcal/mol) Using the Four Sets of Fixed Atomic Charges (See the “Methods” Section)^a^

	exp.	AM1/BCC	RESP/HF/6–31G*	RESP-QM/MM	ABCG2
2PR	–6.40	–3.00 ± 0.14	–3.32 ± 0.08	–4.40 ± 0.09	–5.85 ± 0.18
3MP	–4.77	0.41 ± 0.03	–2.28 ± 0.04	–4.21 ± 0.05	–4.07 ± 0.04
ASP	–9.94	–9.37 ± 0.24	–6.85 ± 0.05	–8.29 ± 0.06	–8.41 ± 0.07
CYC	–4.59	–0.41 ± 0.09	–4.31 ± 0.07	–5.64 ± 0.07	–6.60 ± 0.11
DIA	–5.74	–11.04 ± 0.65	–10.62 ± 0.13	–10.23 ± 0.16	–10.84 ± 0.14
IMI	–9.63	–1.64 ± 0.03	–6.65 ± 0.03	–10.94 ± 0.04	–9.10 ± 0.03
KET	–10.78	–10.28 ± 0.44	–9.23 ± 0.07	–9.06 ± 0.06	–9.65 ± 0.09
NIT	–7.98	–11.75 ± 0.21	–13.89 ± 0.10	–13.85 ± 0.09	–9.33 ± 0.09
PRO	–2.45	–3.45 ± 0.18	–6.42 ± 0.09	–6.45 ± 0.08	–4.08 ± 0.08
TER	–11.14	–15.40 ± 0.98	–9.91 ± 0.07	–13.65 ± 0.08	–13.71 ± 0.08
TIO	–1.40	0.08 ± 0.04	–1.12 ± 0.04	0.14 ± 0.03	–1.06 ± 0.04

a Experimental data are taken from the FreeSolv database.^84^ AM1/BCC data are taken from ref 33, except for IMI, TIO, and 3MP.

**Table 2:** Experimental and Calculated Solvation Free Energies (kcal/mol) in 1-Octanol Using the Four Sets of Fixed Atomic Charges (See the “Methods” Section)^a^

	exp.	AM1/BCC	RESP/HF/6–31G*	RESP-QM/MM	ABCG2
2PR	–6.51	–4.82 ± 0.14	–3.49 ± 0.09	–4.80 ± 0.26	–5.43 ± 0.24
3MP	–6.41	–4.39 ± 0.05	–4.01 ± 0.09	–5.67 ± 0.16	–5.29 ± 0.16
ASP	–11.57	–11.44 ± 0.34	–7.59 ± 0.14	–9.77 ± 0.28	–9.43 ± 0.19
CYC	–6.63	–4.36 ± 0.11	–4.45 ± 0.10	–7.35 ± 0.39	–8.26 ± 0.33
DIA	–12.44	–20.07 ± 0.42	–15.19 ± 0.36	–18.17 ± 0.46	–17.85 ± 0.31
IMI	–9.52	–3.90 ± 0.06	–2.49 ± 0.04	–9.93 ± 0.43	–8.57 ± 0.44
KET	–15.05	–15.12 ± 0.50	–11.30 ± 0.25	–14.30 ± 0.42	–14.13 ± 0.45
NIT	–13.05	–19.79 ± 0.50	–15.50 ± 0.32	–18.83 ± 0.42	–17.60 ± 0.34
PRO	–10.09	–14.12 ± 0.26	–14.22 ± 0.41	–15.18 ± 0.32	–14.88 ± 0.20
TER	–13.73	–18.64 ± 0.40	–10.62 ± 0.15	–18.88 ± 0.74	–17.29 ± 0.30
TIO	–3.88	–3.74 ± 0.04	–3.57 ± 0.08	–3.82 ± 0.06	–3.85 ± 0.06

a Experimental data are derived from the octanol-water Log*P* values reported in the PUBCHEM database^85^ and from the FreeSolv database.^84^ AM1/BCC data are taken from ref 33, except for IMI, TIO, and 3MP.

For the solvation energy in 1-octanol (Δ*G*_0_) reported in Table 2, the general trend observed in water is confirmed with performances increasing in the series AM1/BCC, RESP/HF/6–31G*, RESP-QM/MM, ABCG2. As seen for the Δ*G*_w_, the 1-octanol solvation free energies of the polyfunctional compounds TER, PRO, NIT, and DIA are significantly overestimated for all fixed-charge protocols, thus likely affording an error compensation effect when evaluating water-octanol transfer free energies. On the other hand, it is worth noticing that the AM1/BCC model (as seen for Δ*G*_w_) severely underestimates Δ*G*_0_ for IMI, 3MP and CYC, hence once again positively affecting, as we shall see further on, the corresponding Log*P* by error compensation.

In Figure 4 we report, the correlation plots between experimental and calculated hydration and 1-octanol-solvation free energies, obtained using the four fixed-charge models. Error bars for the calculated data are, on average, below 0.3 kcal/mol (except for the AM1-BCC model taken from ref 33. See Tables 1 and 2) and are not reported on the plot for clarity. Detailed metrics for the four fixed-charge approaches in reproducing the hydration free energy and 1-octanol solvation free energy are presented in Table 3.

**Figure 4:**
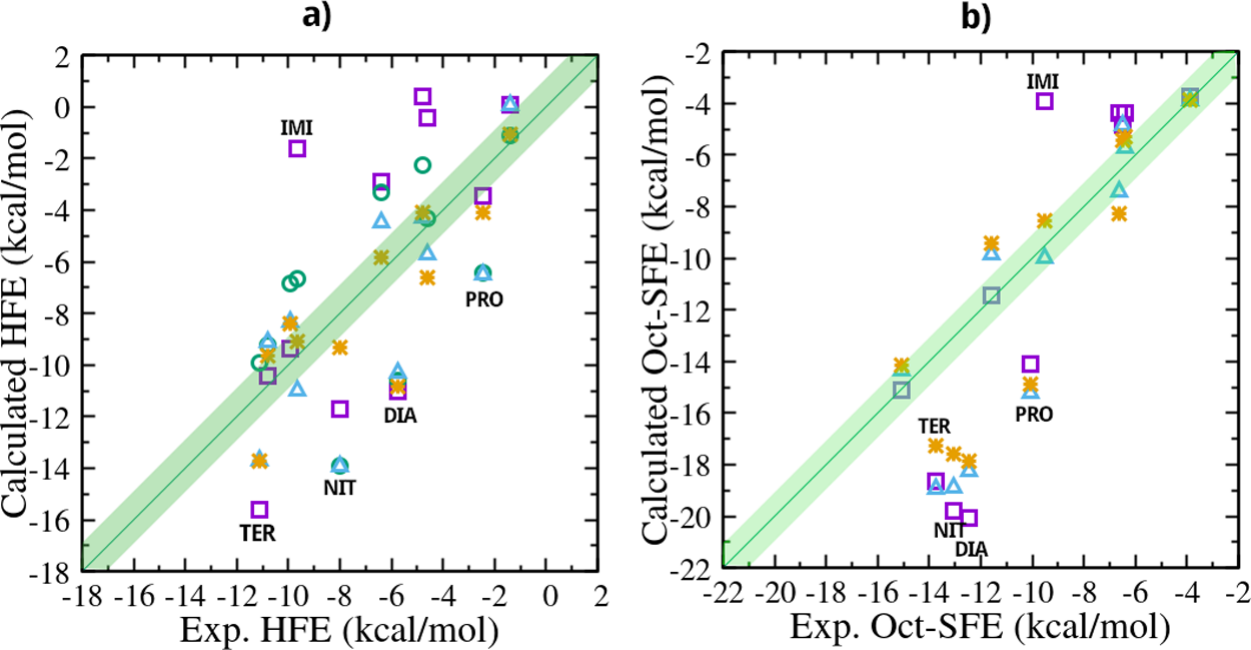
Experimental-calculated correlation plots for the hydration free energy (HFE) Δ*G*_w_ (a) and for the solvation free energy in 1-octanol (SFE) Δ*G*_0_ (b) using the AM1/BCC atomic charges (violet square), the RESP/HF/6–31G* atomic charges (green circles), the RESP-QM/MM atomic charges (cyan triangles), and the ABCG2 atomic charges (orange stars).

**Table 3:** Metrics for Experimental-Calculated Solvation Energies in Water (Δ*G*_w_) and in 1-Octanol (Δ*G*_o_) Using the Four Fixed-Charge Models^a^

Δ*G*_w_							
model	CCC	ρ	*a*	*b*	MUE	τ	MSE
AM1/BCC	0.58	0.68	1.15	1.83	3.43	0.49	–0.79
RESP/HF/6–31G*	0.57	0.58	0.67	–2.20	2.70	0.49	–0.02
RESP-QM/MM	0.70	0.75	0.96	–1.36	2.43	0.49	1.07
ABCG2	0.81	0.84	0.90	–1.42	1.59	0.60	0.72

Δ*G*_0_							
model	CCC	ρ	*a*	*b*	MUE	τ	MSE
AM1/BCC	0.70	0.86	1.64	5.26	3.20	0.56	1.05
RESP/HF/6–31G*	0.67	0.75	1.06	2.09	3.19	0.53	–1.50
RESP-QM/MM	0.74	0.88	1.40	2.37	2.54	0.75	1.62
ABCG2	0.78	0.87	1.28	1.56	2.38	0.71	1.24

a CCC: concordance correlation coefficient; ρ: Pearson correlation coefficients; a: slope of the best line fit; b: best line intercept (kcal/mol); MUE: mean unsigned error (kcal/mol); τ: Kendall rank coefficient; MSE: mean signed error (kcal/mol).

Noticeably, correlation for the solvation free energy in 1-octanol, as measured by the Pearson and Kendall correlation coefficients ρ and τ (see Table 3), is significantly higher than that obtained for the free hydration energy for AM1/BCC, HF/6–31G* and QM/MM (except for ABCG2, where ρ is slightly better in water). Consequently, on average, deviations from the best fitting line are more important for Δ*G*_w_ than they are for Δ*G*_0_, while accuracy (measured by the mean unsigned error (MUE) and depending on how close the best-fitting line is to the 45° line passing through the origin) is, in general, worse for Δ*G*_0_, irrespective of the adopted fixed-charge scheme. So, with few exceptions, we observe best-fitting line slopes closer to 1 and smaller MUE for Δ*G*_w_ compared to Δ*G*_0_ (for which, in contrast, ρ is better).

The two correlation plots exhibit outliers that are common for most fixed-charge approaches, namely NIT, PRO, DIA, and TER, with *both* the Δ*G*_w_ and the Δ*G*_0_ being overestimated. From Figure 4, ABCG2 (orange stars) visibly emerges as the most reliable method for predicting solvation free energies. This is confirmed by the metrics data reported in Table 3. Agreement with the experiment is best measured using the correlation concordance coefficient (CCC),^87^ a metric that combines the accuracy, as the expected squared perpendicular deviation or RMSE from a 45° line through the origin, with correlation, measured by the Pearson Correlation coefficient. As shown in Table 3, ABCG2 consistently produces the best CCC in Δ*G*_w_ and Δ*G*_0_, compared to the other fixed-charge approaches, although it falls short of reaching the performance obtained in a recent test^35^ in large databases of small monofunctional molecules. In this regard, is it worth noting that if we exclude the four polyfunctional outliers NIT, TER, DIA, and PRO, the CCC for ABCG2 raises to 0.93 for both Δ*G*_w_ and Δ*G*_0_, consistently with the results obtained by He et al. on the FreeSolv^84^ and Minnesota solvation^88^ databases, where the weight of bulky compounds is marginal^35^ (see Section “Discussion” further on).

In Figure 5 we show the cross-correlation according to the CCC between the four sets of fixed-charge protocols for Δ*G*_w_ and Δ*G*_0_. Not surprisingly (see Figure 5a), the Δ*G*_w_’s computed with the AM1/BCC protocol are well correlated to those obtained with the HF/6–31G* protocol (CCC = 0.79), given the fact that AM1/BCC charges are supposed to emulate the RESP/HF/6–31G* charges. The CCC decreases to 0.71 when comparing the Δ*G*_w_ computed using the AM1-BCC model with the Δ*G*_w_ evaluated adopting the RESP-QM/MM protocol, that takes into account the explicit solvent polarization due to MM water on the QM solute, and with the Δ*G*_w_ calculated using ABCG2, a model trained also on experimental Δ*G*_w_ of simple solutes (CCC = 0.72).^34^ Remarkably, ABCG2 and RESP-QM/MM hydration free energies are very well correlated (CCC = 0.89) despite the fundamental differences in the two computational protocols.

**Figure 5:**
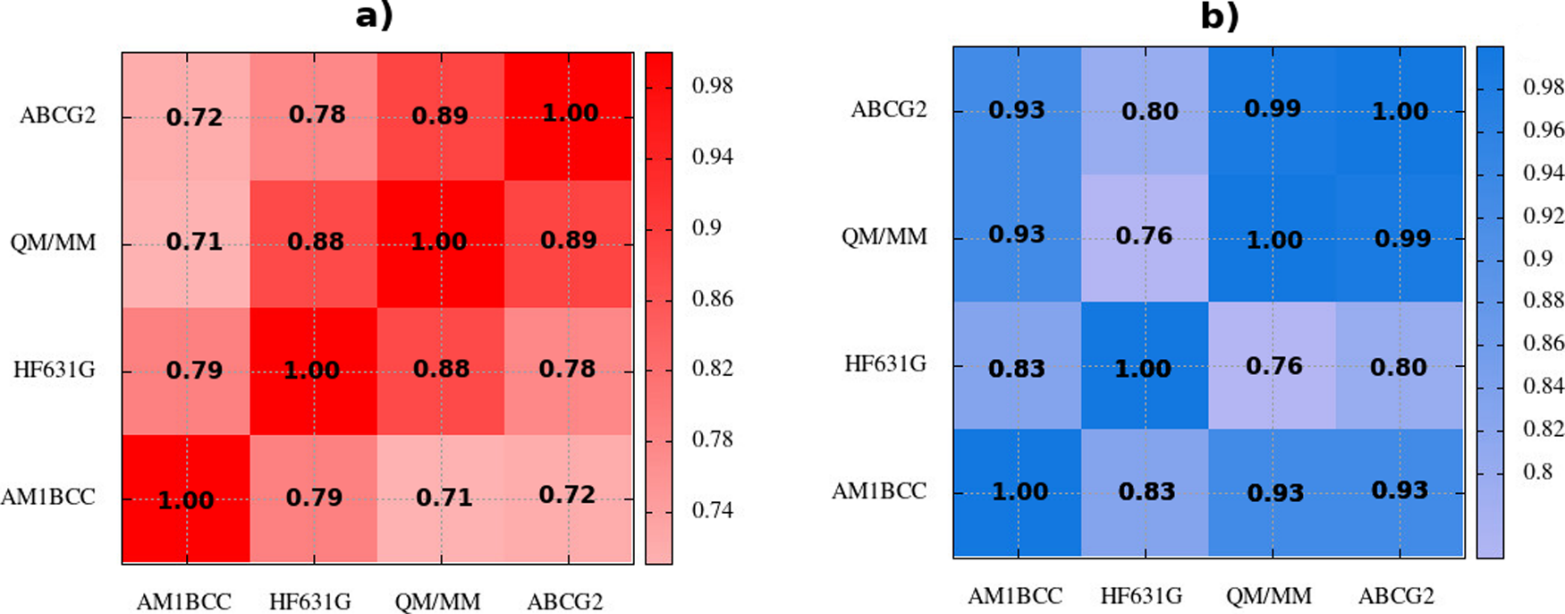
Correlation matrix among the four fixed-charge models for Δ*G*_w_ (a) and Δ*G*_0_ (b).

For Δ*G*_0_ (see Figure 5b), the mutual concordance correlation coefficient among the various fixed-charge protocols is, on average, better than that found for Δ*G*_w_. This is due to the fact that in 1-octanol, the major contribution to the solvation free energy is given by the Lennard-Jones work and not by the recharging work as in water, and, as previously outlined, all four protocols share the same Lennard-Jones parametrization. For Δ*G*_0_, as observed for Δ*G*_w_, the concordance coefficient between the QM/MM and the ABCG2 protocols is the highest (CCC = 0.99). On the other hand, Δ*G*_0_ computed using AM1/BCC appears to be less correlated to the RESP/HF-6–31G* counterpart, compared to ABCG2 and RESP-QM/MM. The excellent CCC between ABCG2 and RESP-QM/MM for the solvation free energies found in water and in 1-octanol is indeed remarkable. The two fixed-charge approaches are consistently providing the best results for both Δ*G*_0_ and Δ*G*_w_, indicating that the cost-effective ABCG2 model is somehow able to capture the main features of atomic charges distribution in condensed phases calculated at the RESP-QM/MM level.

### Log*P* Calculations

In Table 4, we report the 1-octanol–water partition coefficients obtained from the data reported in Tables 1 and 2 computed according to the equation:

$$\mathrm{Log}\;P=\frac{\Delta G_{w}-\Delta G_{o}}{RT\ln(10)}$$ 3

Note that the transfer free energy of the solutes from 1-octanol to water is obtained, in kcal/mol, by multiplying the Log*P* by 1.37. As expected, the effect of the error compensation on the Log*P* and on the transfer free energy is evident for all four examined fixed-charge protocols, both in accuracy and in precision.

**Table 4:** Experimental (Taken from PUBCHEM^85^) and Calculated 1-Octanol-Water Partition Coefficients Log*P* According to the Four Sets of Fixed-Charge Models

	exp.	AM1/BCC	RESP/HF/6–31G*	RESP-QM/MM	ABCG2
2PR	0.08	1.36 ± 0.20	0.12 ± 0.12	0.29 ± 0.28	–0.31 ± 0.30
3MP	1.20	3.50 ± 0.06	1.26 ± 0.10	1.07 ± 0.17	0.89 ± 0.16
ASP	1.19	1.57 ± 0.44	0.54 ± 0.15	1.08 ± 0.29	0.74 ± 0.20
CYC	1.49	3.50 ± 0.14	0.10 ± 0.12	1.25 ± 0.40	1.21 ± 0.35
DIA	4.89	5.42 ± 0.96	3.34 ± 0.38	5.80 ± 0.49	5.12 ± 0.34
IMI	–0.08	1.64 ± 0.06	–3.04 ± 0.05	–0.74 ± 0.43	–0.39 ± 0.44
KET	3.12	3.38 ± 0.68	1.51 ± 0.26	3.82 ± 0.42	3.27 ± 0.46
NIT	3.70	5.92 ± 0.52	1.18 ± 0.34	3.63 ± 0.43	6.04 ± 0.35
PRO	5.58	7.77 ± 0.36	5.69 ± 0.42	6.37 ± 0.33	7.88 ± 0.22
TER	1.89	2.19 ± 0.41	0.52 ± 0.17	3.82 ± 0.74	2.61 ± 0.31
TIO	1.81	2.79 ± 0.06	1.79 ± 0.09	2.89 ± 0.07	2.04 ± 0.07

In Figure 6 we report the correlation plot (CCC) between experimental and calculated Log*P* alongside with the mutual correlation among the four fixed-charge protocols. Accuracy and precision metrics for calculated vs experimental Log*P* are reported in Table 5. From Figure 6a, the general improvement for the Log*P* compared to the solvation free energies (see Figure 4) is evident, showing the effectiveness of the error compensation due to the systematic overpolarization for NIT, PRO, TER, and DIA in the four protocols and to the underpolarization of IMI, 3MP, and CYC observed in AM1/BCC. The MUE on the transfer free energy is well below 1.5 kcal/mol in all cases with a Pearson correlation coefficient ranging from 0.87 for RESP/HF/6–31G* to 0.97 for ABCG2.

**Figure 6:**
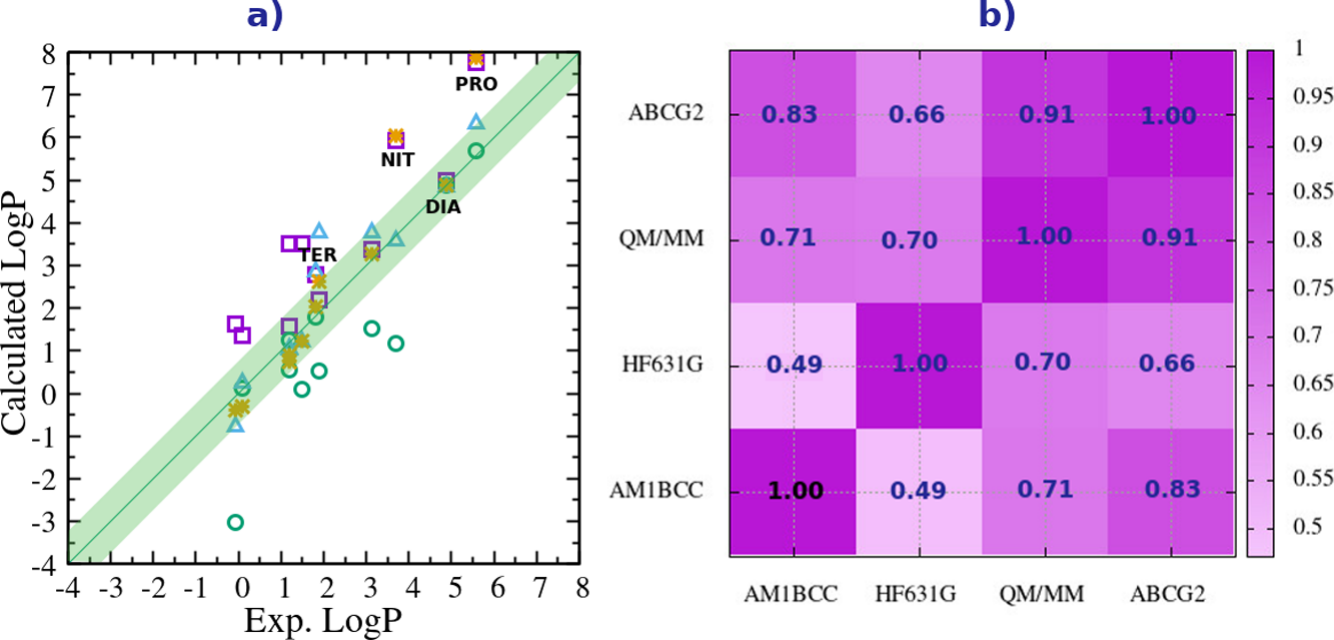
(a) Experimental-calculated water-octanol partition coefficients (Log*P*) using the AM1/BCC atomic charges (violet square), the RESP/HF/6–31G* atomic charges (green circles), the RESP-QM/MM atomic charges (cyan triangles), and the ABCG2 atomic charges (orange stars). (b) Matrix of concordance correlation coefficient (CCC) among the four fixed-charge models for the Log*P*.

**Table 5:** Metrics for Experimental-Calculated Log*P* Using the Four Fixed-Charge Models^a^

Δ*G*_w_							
model	CCC	ρ	*a*	*b*	MUE	τ	MSE
AM1/BCC	0.73	0.90	0.98	1.30	1.25	0.62	–1.25
RESP/HF/6–31G*	0.74	0.87	1.01	–1.10	1.12	0.64	1.08
RESP-QM/MM	0.92	0.95	1.17	0.01	0.62	0.89	–0.40
ABCG2	0.89	0.97	1.41	–0.54	0.70	0.96	–0.38
ABCG2^b^	0.97	0.98	1.15	–0.30	0.34	1.00	0.04

a ρ: Pearson correlation coefficients; *a*: slope of the best line fit; *b*: best line intercept (kcal/mol); MUE: mean unsigned error; τ: Kendall rank coefficient; MSE: mean signed error.

b Metrics computed excluding the outliers NIT and PRO.

The best accuracy and precision as measured by the CCC (see Table 5) is obtained by the expensive protocol based on RESP-QM/MM (CCC = 0.92), closely followed by the cost-effective ABCG2 model with CCC = 0.89. As observed for the solvation free energies, Figure 6b shows that the protocols RESP-QM/MM and ABCG2 yield strongly correlated (0.91) Log*P* predictions. Interestingly, while the compensation error in ABCG2 provides a nearly optimal Log*P* value for DIA, ABCG2-computed Log*P* for the bulky and flexible compounds NIT and PRO are still significantly overestimated by ≃2.3 Log*P* units (corresponding to 3.1 kcal/mol for the transfer free energy), remaining similar to those computed with the precursor AM1/BCC model. If we eliminate the NIT and PRO outliers, the CCC for the remaining 9 compounds obtained with ABCG2 raises to a stunning 0.97 value with a MUE as low as 0.34, corresponding to an error on the transfer free energy of less than 0.5 kcal/mol. It is also worth noting that the proposed RESP-QM/MM approach for fixed-charge determination (yielding the best CCC on the 11 compounds), does not produce any significant outlier, projecting this methodology (based on averaging the RESP atomic charges on solute conformations extracted from a QM/MM simulation) as the best alternative (in case of ABCG2 failure) for fixed-charge determination when dealing with bulky and polyfunctional compounds as well as simple monofunctional molecules.

We have seen that the good-to-excellent performances of the fixed-charge models in reproducing experimental water-octanol transfer free energies is many cases due to an error compensation, whereby solvation free energies are overestimated in both the polar and hydrophobic environment. Past and recent results on protein data sets have provided consistent statistical insights into the hydrophobic/hydrophilic nature of druggable pockets. In ref 89, for example, it was shown that druggable sites in proteins are mostly delimited by hydrophobic residues. The same conclusion was drawn in an extended survey,^90^ where more than 70% of the analyzed protein pockets were classified as mostly hydrophobic. Hydrophobicity score of protein binding pockets can today be assessed using several popular public web-based tools such as Ichem,^91^ sc-PDB^92^ or PockDrug,^93^ to name a few. Based on such well-established consensus on the prevalent hydrophobic nature of the druggable sites in proteins, we can thus reasonably expect that simple fixed-charge models such as the ABCG2 protocol can deliver, via alchemical simulations, transfer free energies from water to the mostly hydrophobic environment of druggable binding sites with accuracy comparable to that observed in the Log*P*, where the compensation error play a major role. Verification of this inference is clearly beyond the scope of the present contribution as it would require a dedicated study, carefully selecting other ligand molecules and host systems (proteins or cavitand) reflecting the average chemical nature of known binding pockets in protein.

### Impact of Fixed-Charge Set Selection on Computed Solvation Free Energies

As discussed in the “Methods” section, atomic charges in all four cases have been averaged over 10 conformations sampled in the 20 ps-long QM/MM simulations of the compounds in water. AM1-BCC or ABCG2 atomic charges are, in general, rather insensitive to the molecular conformation^94^ as they are obtained from the density-based Mulliken charges corrected by BCC. While Mulliken atomic charges are mostly sensitive to the *local* environment of an atom, e.g., to bond distances or bending angles that are optimized by default in the antechamber generation, bond charge corrections, by design, are independent from the conformation, as they are based on the atomic types involved in the molecular bonds. The small charge variability across the ten conformations for the ABCG2 model can be appreciated in the SI, in Tables S12–S33.

In case of the RESP-QM/MM model, conformation dependency of the RESP charges should be handled with due care. RESP charges, since they are designed to fit the electrostatic potential around the molecule, are very sensitive to molecular conformation, especially when the dipole or quadrupole moments change significantly from one conformer to the other. Most of the molecules in the present set are relatively rigid with basically minor conformational variability. However, dialifor in water stands out as a remarkable exception; in fact, as we shall detail further, it exhibits a competition between an extended (with low dipole) and compact (with a high dipole) conformation, with a strong prevalence of the latter. As detailed in the “Methods” section, the 10 conformations of dialifor for fixed charge averaging were sampled in a short 20 ps QM/MM simulation. While 20 ps are enough to catch the charge modulations due to local bond and bending changes and to water molecule interactions (e.g., formation of transient H-bonds), they are largely insufficient to allow exchanges between structurally disparate conformers. In fact, all the 10 dialifor conformations sampled during the QM/MM simulations belong to the compact structures, hence yielding a set of fixed RESP-QM/MM atomic charges, [*Q*_*c*_], for the compact conformation alone. The small RESP-QM/MM charge variation detected in the 10 conformers (see SI Table S20) is most likely due to the fact that they all belong to the compact structure. Sampling the less likely extended conformation using a much longer QM/MM MD simulation would be exceedingly costly. We hence performed a Hamiltonian Replica Exchange simulation of ABCG2 dialifor in water using the same setup that we employed in the QM/MM simulations, namely NVT ensemble, same MD box and same number of SPC/E water molecules. The HREM setup involves the solute tempering of dialifor alone (water is kept cold) with 8 replica states and a scaling protocol with a maximum scaling factor of 0.1, corresponding to a solute “temperature” of 3000 K. HREM of dialifor in water was conducted for 6 ns using the ORAC program^59^ on the CRESCO6-HPC cluster^95^ yielding an average optimal acceptance ratio of 30% between contiguous replica states.

In Figure 7 we show the histogram of the C4–C22 distance distribution (see Figure S5 of Supporting Information for atomic label) as a probe for the compact conformation (with the oxo-ethyl group stacked on the dioxo-isoindole moiety) and the extended conformation. We found a ratio of ≃2 between the compact and extended conformations. From the set of the extended conformations, we extracted 10 random conformations, which were fed to the CP2K program for single-point RESP atomic charges calculations. The resulting 10 sets of atomic charges were then averaged to obtain the [*Q*_*e*_] fixed RESP-QM/MM atomic charges for the elongated structure.

**Figure 7:**
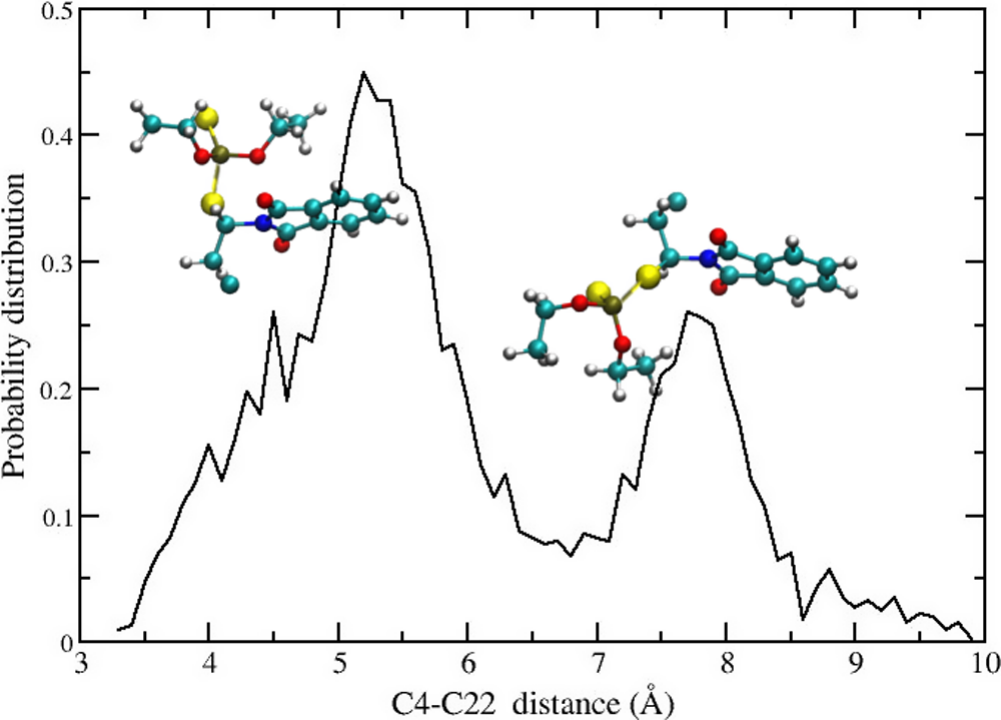
Probability distribution of the C4–C22 distance marking the compact and extended structures in dialifor.

In Figure 8 we report the RESP-QM/MM charges obtained by averaging over 10 conformations of the extended (red curve) and the compact (black curve) conformers of dialifor. As can be seen, differences are significant, especially on the CH_3_ and CH_2_ groups and on the carboxylate moiety, highlighting the sensitivity of the RESP procedure to conformational changes.

**Figure 8:**
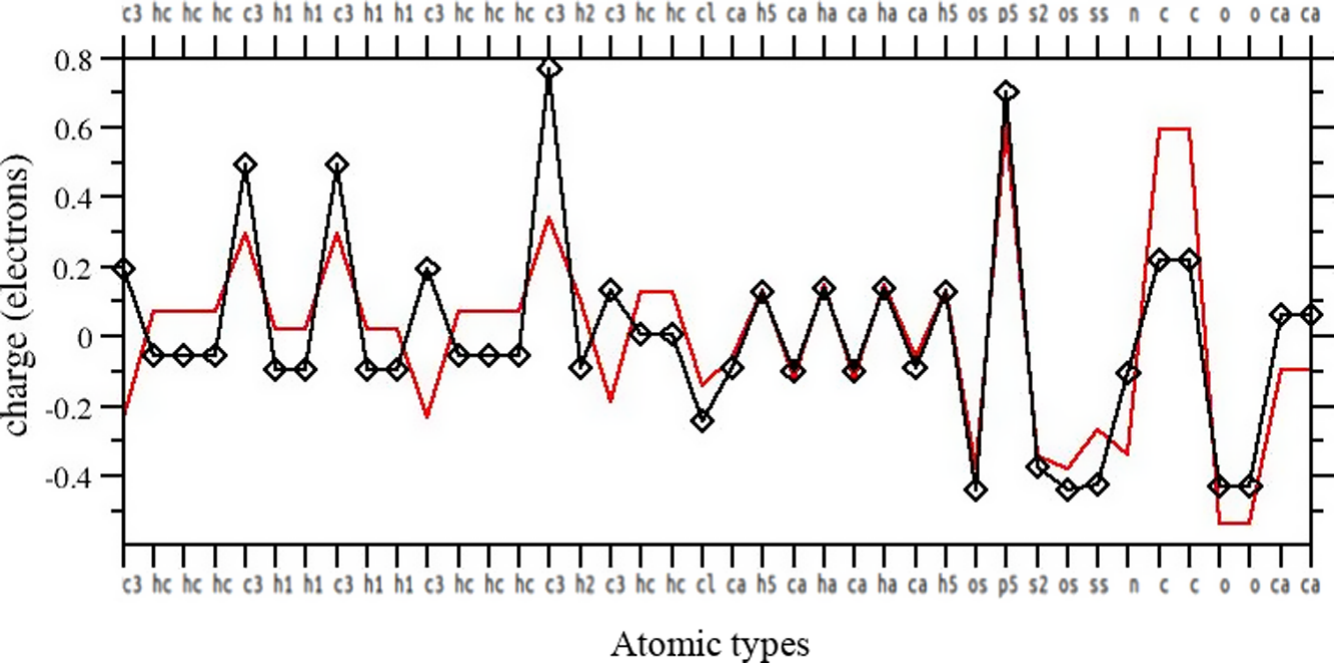
RESP-QM/MM atomic charges in dialifor for the elongated (red) and compact (black) structures.

We hence determined via fast-growth alchemy the solvation free energy of dialifor in water and in 1-octanol using the [*Q*_e_] set and a set of RESP-QM/MM atomic charges obtained by combining the average charge [*Q*_e_] and [*Q*_c_] with the appropriate weigh factor desumed from the HREM simulation, i.e. *Q*_c/e_^*i*^ = 0.67*Q*_c_^*i*^ + 0.33*Q*_e_^*i*^, where the *i* index runs on the atoms of the molecule. The results are shown in Table 6.

**Table 6:** Solvation Free Energies (in kcal/mol) via Fast-Growth of Dialifor in Water (Δ*G*_w_), 1-Octanol (Δ*G*_o_), and Water-Octanol Transfer Free Energy (Δ*G*_w–o_) Calculated with Different RESP-QM/MM Atomic Charges Sets (See Text)^a^

	Δ*G*_w_	Δ*G*_o_	Δ*G*_w–o_
[*Q*_c_]	–10.23 ± 0.16 (−5.74)	–18.17 ± 0.46 (−12.44)	7.94 ± 0.67 (6.70)
[*Q*_e_]	–11.65 ± 0.13 (−5.74)	–18.01 ± 0.37 (−12.44)	6.36 ± 0.54 (6.70)
0.67[*Q*_c_] + 0.33[*Q*_e_]	–10.48 ± 0.13 (−5.74)	–17.98 ± 0.47 (−12.44)	7.50 ± 0.67 (6.70)

a In parentheses are reported the corresponding experimental values.

Despite the important charge variations in the [*Q*_e_] and [*Q*_c_] sets (see Figure 8), significant differences can only be seen for the case of the hydration free energy, where the extended charge set [*Q*_e_] yields a Δ*G*_w_ which is lower by ≃1.4 kcal/mol compared to that obtained with the [*Q*_c_]. Minor changes are observed for the solvation free energies in 1-octanol. The compensation error in adjusting the water-octanol transfer free energy, Δ*G*_w–o_, continues to play a major role for any atomic charge set.

## Discussion

Recently, ABCG2 in combination with GAFF2 has been extensively tested on the FreeSolv and Minnesota solvation database of mostly monofunctional organic molecules of low molecular weight using an MD-based alchemical approach with explicit solvent. ABCG2-computed solvation free energies showed a decisive improvement compared to the AM1/BCC/GAFF2 model.^35^ Outliers were found mostly for compounds with a large molecular weight and/or including phosphorus or sulfur atoms, accounting for a small percentage of the total tested solute molecules. In Figure 9, we show the distribution of the number of heavy atoms, *N*_h_, in the FreeSolv and Minnesota databases. Both distributions exhibit an intense peak at *N*_h_ = 8 with less than 10% of the compounds with *N*_h_ > 12 and less than 2% with *N*_h_ > 20. In our case, more than half of the tested 11 compounds have high molecular weight and *N*_h_ > 12, i.e. in a range that was poorly sampled in the He et al. study (see Figure 1D of ref 35). As Figure 4 shows, the large weight of polyfunctional and bulky compounds such as NIT, TER, DIA, and PRO, is responsible for the significantly lower ρ (0.84) observed in our limited batch of molecules for Δ*G*_w_ in comparison to the corresponding value (ρ = 0.98) obtained by He et al. (see Table 2 of ref 35). On the other hand, the high Pearson coefficient ρ for the ABCG2-computed Δ*G*_w_ in ref 35 is a direct consequence of the low incidence of bulky and polyfunctional molecules in the FreeSolv and Minnesota databases.

**Figure 9:**
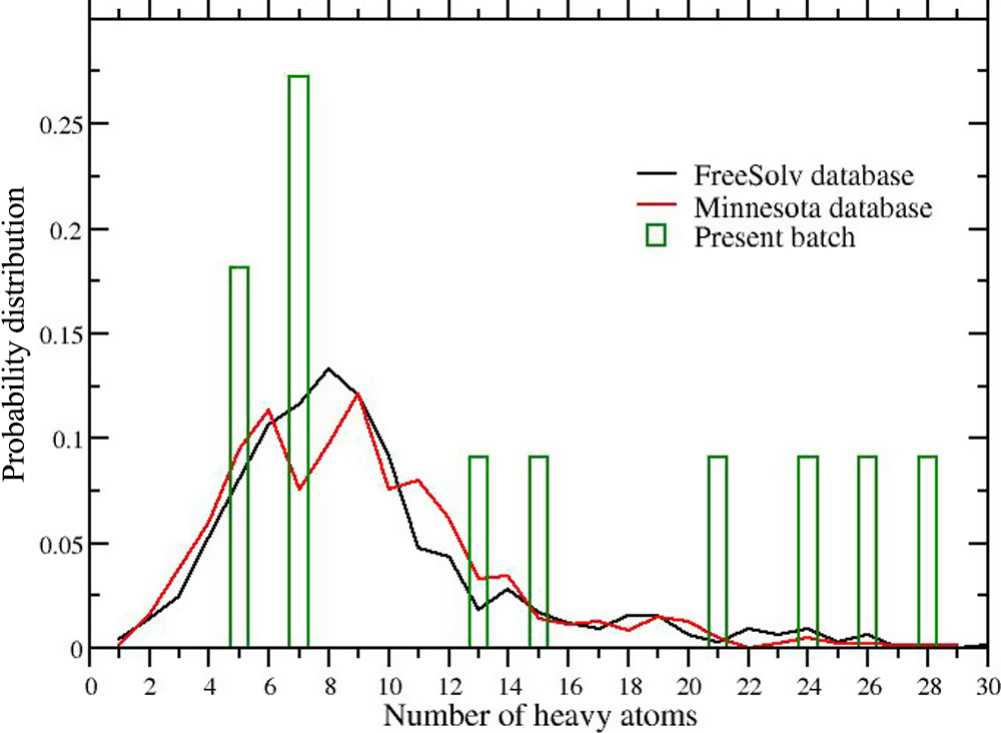
Distribution of the number of heavy atoms (*N*_h_) in the Minnesota (red line) and FreeSolv (black line) databases. The histogram refers to the distribution of the number of heavy atoms for the set of compounds of Figure 1.

In our case, when we focus on the water-octanol transfer free energy, the Pearson coefficient ρ obtained with ABCG2 increases up to 0.97 (see Table 5) and the MUE drops from 1.59 and 2.38 kcal/mol for Δ*G*_w_ and Δ*G*_0_, respectively, to only 0.95 kcal/mol for the transfer free energy. Such error compensation in computing the transfer free energies in our batch of compounds has already been previously highlighted.^33^ A similar error compensation is observed for the 201 compounds (see Table 3 of ref 35) taken from the FreeSolv database. With ABCG2, such a subset yielded a MUE of 1.01 kcal/mol for Δ*G*_w_, that decreased to just 0.63 kcal/mol for the octanol–water transfer free energy.

The improvement of the ABCG2 model in computing solvation and transfer free energies when compared to the precursor AM1/BCC is exclusively due the new set of bond charge corrections on the fixed atomic charges of the solute involving mostly non carbon heavy atoms (see Figure 2), as the remainder of the potential parametrization is unchanged in the two protocols sharing the same GAFF2 parameter set for the bonded part and the same Lennard-Jones atom–atom parameters. This outcome underlines the crucial importance of the fixed-charge parametrization and electrostatic interactions, in general, in devising effective potential models for computational drug design.

Remarkably, the empirical and cost-effective ABCG2 model provides water-octanol transfer free energies that are comparable in accuracy to those computed using the expensive RESP-QM/MM atomic charges protocol, accounting for the average polarization by explicit MM water on a quantum solute in standard condition via QM/MM simulations. While this match can be in part fortuitous, the low-cost fixed-charge ABCG2 model appears to effectively capture the physics regulating the electrostatic interactions in fully atomistic models simulated in standard conditions, thus encouraging its use in MD-based high-throughput screening in drug discovery projects.

## Conclusions

In this paper we have compared the performances of four different fixed-charge protocols on the calculation, via classical molecular dynamics simulation (MD) with explicit solvent, of solvation and transfer free energies for a challenging batch of mostly polyfunctional solute molecules characterized by complex conformational landscapes. Solvation free energy were computed using an efficient and reliable fast-growth alchemical MD approach, based on a combination of Hamiltonian replica exchange sampling and nonequilibrium alchemical solute recoupling trajectories.

The fixed-charge protocols examined in this study include the popular AM1/BCC and RESP/HF/6–31G* approaches, the recently proposed ABCG2 model, and an expensive technique based on QM/MM sampling of the QM solute in MM water. The ABCG2 model emerges as the best alternative among the four protocols for the hydration free energy and 1-octanol solvation free energy, yielding correlation concordance coefficients of 0.81 and 0.78, respectively, significantly outperforming the precursor AM1/BCC. As a result of a systematic error compensation, the correlation metrics in ABCG2 decisively improves when evaluating the water-octanol transfer free energy, with correlation concordance coefficient reaching 0.9 and a mean unsigned error of less than 1 kcal/mol.

The improvement observed for ABCG2 with respect to its precursor AM1/BCC model is due exclusively to the new set of bond charge corrections, highlighting the importance of the modeling of electrostatic interactions when computing transfer free energies of drug-like molecules involving two environment with disparate chemical-physical properties.

The cost-effective ABCG2 model is found to be comparable in accuracy and precision to the far more expensive approach based RESP-QM/MM derived from QM/MM simulations. Given that the binding free energy is in essence a transfer free energy of a drug-like molecule from the bulk solvent to the protein environment, the performance of the simple ABCG2 model holds great promise for high-throughput in silico prediction of ligand-protein binding free energies in drug discovery projects.

## Supplementary Material



## Data Availability

Gromacs/ORAC input files, work data, and ancillary scripts to reproduce the results for solvation free energies of the 11 compounds are available at the general-purpose open-access repository Zenodo (https://zenodo.org/records/15358487).

## Notes

Atomic types and charges for the 11 compounds of the first figure used in this study, obtained with the protocols AM1/BCC, RESP/HF/6–31G*, RESP-QM/MM, and ABCG2 (PDF)
