# Supplementary material for: Solvation Free Energies of Drug-like Molecules via Fast Growth in an Explicit Solvent: Assessment of the AM1-BCC, RESP/HF/6–31G*, RESP-QM/MM, and ABCG2 Fixed-Charge Approaches
Source: J Chem Theory Comput. 2025 Aug 11;21(16):7977–90. doi: 10.1021/acs.jctc.5c00749 (PMC12392459; doi:10.1021/acs.jctc.5c00749)
Supplement: Supplementary file 1 [file ct5c00749_si_001.pdf]

Supporting Information for "Solvation Free Energies of Drug-like Molecules via Fast-growth in Explicit Solvent: Assessment of the AM1-BCC, RESP/HF/6-31G*, RESP-QM/MM, and ABCG2 Fixed-charge Approaches"

Matteo Orlandi,[†,‡] Yiqi Geng,[†] Marina Macchiagodena,[†] Marco Pagliai,[*,†] and Piero Procacci[*,†]

†*Dipartimento di Chimica "Ugo Schiff", Università degli Studi di Firenze, Via della Lastruccia 3, 50019 Sesto Fiorentino, Italy*

‡*Dipartimento di Neuroscienze, Psicologia, Area del Farmaco e Salute del Bambino, Università degli Studi di Firenze, Via Ugo Schiff 6, 50019 Sesto Fiorentino, Italy*

E-mail: marco.pagliai@unifi.it; piero.procacci@unifi.it

1 Atomic Charges

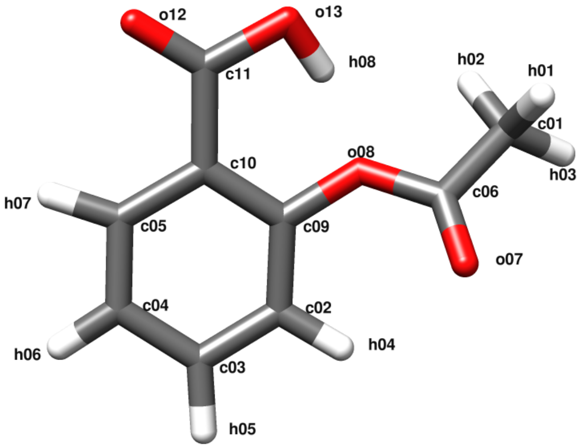

Figure S1: Atoms labels for acetylsalycilic acid.

Table S1: Atomic charges (e) for acetylsalycilic acid. For atoms labels see Figure S1.

Atom type	Atom label	AM1/BCC	RESP/HF/6-31G*	RESP-QM/MM	ABCG2
c3	c01	-0.1526	-0.3688	-0.1341	-0.1521
h1	h01	0.0846	0.1239	0.0655	0.0850
h1	h02	0.0846	0.1239	0.0655	0.0850
h1	h03	0.0846	0.1239	0.0655	0.0850
ca	c02	-0.2102	-0.1896	-0.1286	-0.1307
ha	h04	0.1615	0.1724	0.1448	0.1450
ca	c03	-0.0846	-0.1310	-0.1659	-0.0659
h4	h05	0.1439	0.1591	0.1674	0.1259
ca	c04	-0.1473	-0.1362	-0.0655	-0.1303
ha	h06	0.1451	0.1514	0.1275	0.1269
ca	c05	-0.0599	-0.1804	-0.1683	-0.0438
h5	h07	0.1611	0.1737	0.1332	0.1419
c	c06	0.6502	0.7541	0.6732	0.5989
o	o07	-0.5032	-0.5323	-0.5528	-0.4585
os	o08	-0.5270	-0.3878	-0.3836	-0.3937
ca	c09	0.3981	0.2415	0.2676	0.1475
ca	c10	-0.2470	-0.0970	-0.0963	-0.1827
c	c11	0.7994	0.7027	0.7227	0.6124
o	o12	-0.4934	-0.5113	-0.5806	-0.4539
oh	o13	-0.7278	-0.5864	-0.5773	-0.6113
ho	h08	0.4399	0.3942	0.4201	0.4694

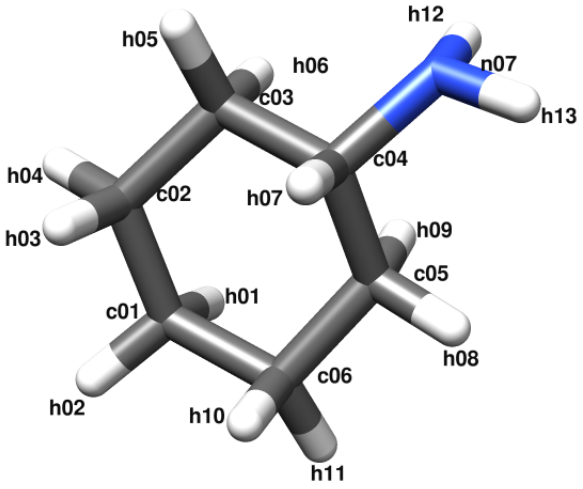

Figure S2: Atoms labels for cyclohexanamine.

Table S2: Atomic charges (e) for cyclohexanamine. For atoms labels see Figure S2.

Atom type	Atom label	AM1/BCC	RESP/HF/6-31G*	RESP-QM/MM	ABCG2
c3	c01	-0.0782	-0.0141	0.0783	-0.0782
hc	h01	0.0395	0.0100	-0.0312	0.0392
hc	h02	0.0395	0.0100	-0.0312	0.0392
c3	c02	-0.0768	-0.0458	0.0381	-0.0748
hc	h03	0.0411	0.0142	-0.0357	0.0402
hc	h04	0.0411	0.0190	-0.0153	0.0402
c3	c03	-0.0735	-0.0984	-0.0138	-0.0900
hc	h05	0.0460	0.0180	-0.0312	0.0425
hc	h06	0.0460	0.0297	-0.0265	0.0425
c3	c04	0.0417	0.4626	0.6087	0.1643
hc	h07	0.0589	-0.0272	-0.1037	0.0481
c3	c05	-0.1145	-0.0984	-0.0138	-0.0900
hc	h08	0.0374	0.0180	-0.0312	0.0425
hc	h09	0.0374	0.0297	-0.0265	0.0425
c3	c06	-0.0719	-0.0458	0.0381	-0.0748
hc	h10	0.0398	0.0142	-0.0357	0.0402
hc	h11	0.0398	0.0190	-0.0153	0.0402
n3	n07	-0.5451	-0.9689	-1.0675	-1.0146
hn	h12	0.2258	0.3270	0.3577	0.4004
hn	h13	0.2258	0.3270	0.3577	0.4004

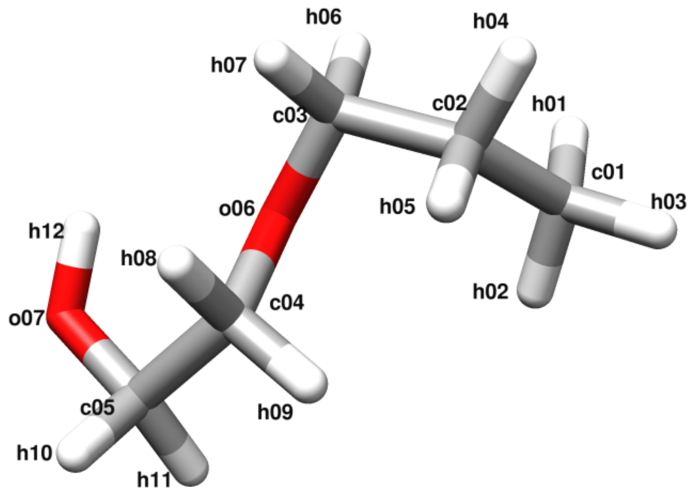

Figure S3: Atoms labels for 2-propoxyethanol.

Table S3: Atomic charges (e) for 2-propoxyethanol. For atoms labels see Figure S3.

Atom type	Atom label	AM1/BCC	RESP/HF/6-31G*	RESP-QM/MM	ABCG2
c3	c01	-0.0935	-0.1574	-0.1075	-0.0911
hc	h01	0.0359	0.0399	0.0232	0.0373
hc	h02	0.0359	0.0399	0.0232	0.0373
hc	h03	0.0359	0.0399	0.0232	0.0373
c3	c02	-0.0852	0.1242	0.1739	-0.1129
hc	h04	0.0539	-0.0205	-0.0271	0.0448
hc	h05	0.0539	-0.0205	-0.0271	0.0448
c3	c03	0.1333	0.0730	0.1758	0.1534
h1	h06	0.0276	0.0421	0.0023	0.0482
h1	h07	0.0276	0.0421	0.0023	0.0482
c3	c04	0.0870	0.1152	0.2337	0.1096
hc	h08	0.0377	0.0291	-0.0100	0.0359
hc	h09	0.0377	0.0291	-0.0100	0.0359
c3	c05	0.1326	0.1940	0.3068	0.1522
h1	h10	0.0521	0.0281	-0.0222	0.0547
h1	h11	0.0521	0.0281	-0.0222	0.0547
os	o06	-0.4377	-0.3758	-0.4560	-0.4744
oh	o07	-0.6003	-0.6370	-0.6874	-0.7206
ho	h12	0.4138	0.3865	0.4048	0.5047

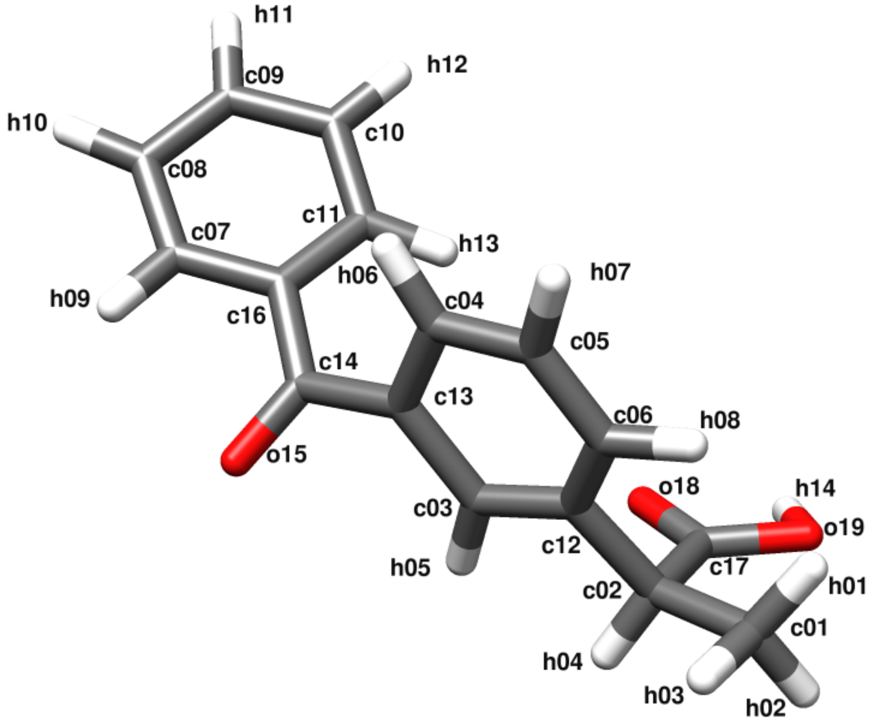

Figure S4: Atoms labels for ketoprofen.

Table S4: Atomic charges (e) for ketoprofen. For atoms labels see Figure S4.

Atom type	Atom label	AM1/BCC	RESP/HF/6-31G*	RESP-QM/MM	ABCG2
c3	c01	-0.0831	-0.2074	0.0364	-0.0903
hc	h01	0.0525	0.0619	-0.0134	0.0520
hc	h02	0.0525	0.0619	-0.0134	0.0520
hc	h03	0.0525	0.0619	-0.0134	0.0520
c3	c02	-0.1069	0.1639	0.3466	-0.0548
hc	h04	0.0811	0.0237	-0.0664	0.0917
ca	c03	-0.0805	-0.1840	-0.2185	-0.0472
h4	h05	0.1513	0.1517	0.1229	0.1358
ca	c04	-0.0851	-0.1300	-0.0918	-0.0680
h4	h06	0.1476	0.1267	0.0910	0.1270
ca	c05	-0.1376	-0.1846	-0.1382	-0.1258
ha	h07	0.1436	0.1537	0.1415	0.1222
ca	c06	-0.0896	-0.1505	-0.1597	-0.0736
h4	h08	0.1487	0.1545	0.1249	0.1362
ca	c07	-0.0916	-0.1414	-0.1261	-0.0667
ha	h09	0.1413	0.1440	0.1054	0.1293
ca	c08	-0.1439	-0.1438	-0.0966	-0.1253
ha	h10	0.1376	0.1321	0.1154	0.1195
ca	c09	-0.1010	-0.1022	-0.1170	-0.0860
ha	h11	0.1365	0.1378	0.1337	0.1170
ca	c10	-0.1424	-0.1438	-0.0966	-0.1253
ha	h12	0.1395	0.1321	0.1154	0.1195
ca	c11	-0.0749	-0.1414	-0.1261	-0.0667
h4	h13	0.1533	0.1440	0.1054	0.1293
ca	c12	-0.1179	0.0223	-0.0043	-0.1039
ca	c13	-0.1634	-0.0172	0.0370	-0.1688
c	c14	0.5386	0.4666	0.3854	0.5893
o	o15	-0.4777	-0.4937	-0.4866	-0.5283
ca	c16	-0.1722	0.0195	0.0612	-0.1696
c	c17	0.7737	0.6165	0.5238	0.5987
o	o18	-0.4875	-0.5649	-0.5561	-0.5068
oh	o19	-0.7260	-0.6091	-0.5861	-0.6432
ho	h14	0.4312	0.4392	0.4643	0.4788

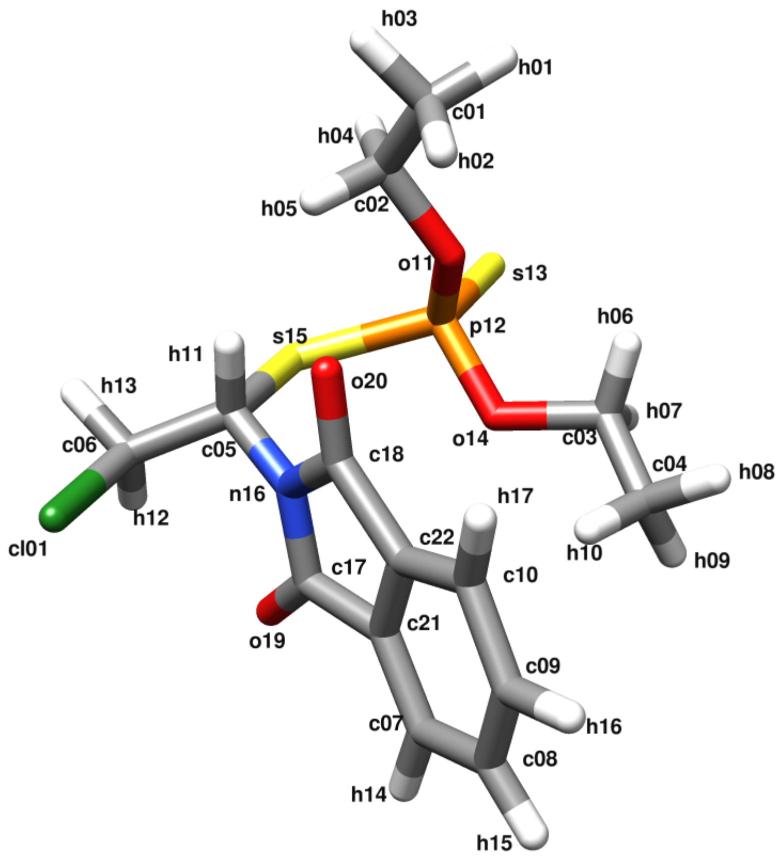

Figure S5: Atoms labels for dialifor.

Table S5: Atomic charges (e) for dialifor. For atoms labels see Figure S5.

Atom type	Atom label	AM1/BCC	RESP/HF/6-31G*	RESP-QM/MM	ABCG2
c3	c01	-0.111	-0.1375	0.1926	-0.1197
hc	h01	0.053	0.0480	-0.0536	0.0569
hc	h02	0.053	0.0480	-0.0536	0.0569
hc	h03	0.053	0.0480	-0.0536	0.0569
c3	c02	0.145	0.2090	0.4927	-0.0299
h1	h04	0.075	0.0302	-0.0965	0.0727
h1	h05	0.075	0.0302	-0.0965	0.0727
c3	c03	0.164	0.2090	0.4927	-0.0299
h1	h06	0.057	0.0302	-0.0965	0.0727
h1	h07	0.057	0.0302	-0.0965	0.0727
c3	c04	-0.123	-0.1375	0.1926	-0.1197
hc	h08	0.059	0.0480	-0.0536	0.0569
hc	h09	0.059	0.0480	-0.0536	0.0569
hc	h10	0.059	0.0480	-0.0536	0.0569
c3	c05	0.313	0.2286	0.7706	0.2705
h2	h11	0.111	0.1011	-0.0916	0.1240
c3	c06	0.010	-0.1147	0.1316	0.1003
hc	h12	0.085	0.1423	0.0047	0.1121
hc	h13	0.085	0.1423	0.0047	0.1121
cl	cl01	-0.136	-0.2080	-0.2442	-0.2609
ca	c07	-0.060	-0.1263	-0.0923	-0.0403
h5	h14	0.162	0.1595	0.1265	0.1438
ca	c08	-0.113	-0.1429	-0.0992	-0.0959
ha	h15	0.146	0.1589	0.1400	0.1294
ca	c09	-0.116	-0.1429	-0.0992	-0.0959
ha	h16	0.146	0.1589	0.1400	0.1294
ca	c10	-0.058	-0.1263	-0.0923	-0.0403
h5	h17	0.161	0.1595	0.1265	0.1438
os	o11	-0.517	-0.3527	-0.4408	-0.3264
p5	p12	1.124	0.6784	0.7031	0.9168
s2	s13	-0.669	-0.4288	-0.3757	-0.4660
os	o14	-0.504	-0.3527	-0.4408	-0.3264
ss	s15	-0.513	-0.3051	-0.4248	-0.4234
n	n16	-0.222	-0.0009	-0.1068	-0.3935
c	c17	0.621	0.2986	0.2179	0.5891
c	c18	0.620	0.2986	0.2179	0.5891
o	o19	-0.530	-0.4349	-0.4316	-0.4768
o	o20	-0.554	-0.4349	-0.4316	-0.4768
ca	c21	-0.133	0.0463	0.0622	-0.1354
ca	c22	-0.136	0.0463	0.0622	-0.1354

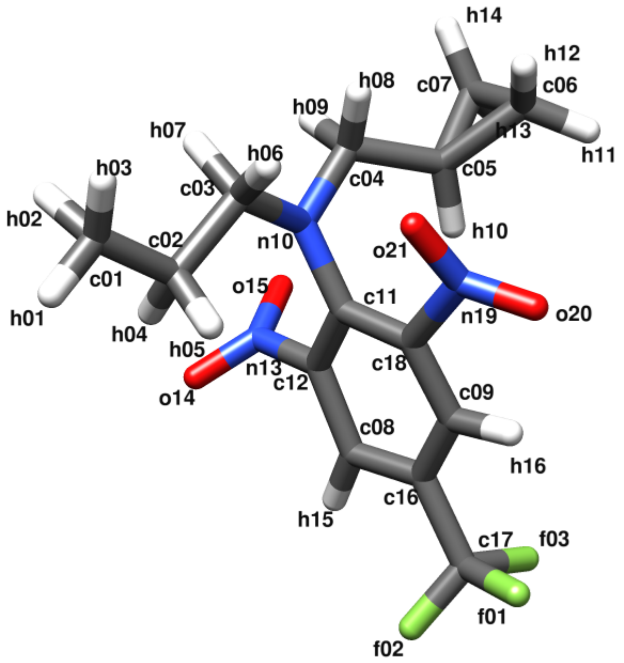

Figure S6: Atoms labels for profluralin.

Table S6: Atomic charges (e) for profluralin. For atoms labels see Figure S6.

Atom type	Atom label	AM1/BCC	RESP/HF/6-31G*	RESP-QM/MM	ABCG2
c3	c01	-0.0931	-0.1907	-0.0172	-0.0943
hc	h01	0.0410	0.0515	0.0033	0.0398
hc	h02	0.0410	0.0515	0.0033	0.0398
hc	h03	0.0410	0.0515	0.0033	0.0398
c3	c02	-0.1007	0.0430	0.1830	-0.1028
hc	h04	0.0528	0.0210	-0.0541	0.0568
hc	h05	0.0528	0.0210	-0.0541	0.0568
c3	c03	0.2173	-0.0834	0.1324	0.2214
h1	h06	0.0656	0.0904	-0.0138	0.0606
h1	h07	0.0656	0.0904	-0.0138	0.0606
c3	c04	0.2500	-0.0461	0.2151	0.2410
h1	h08	0.0618	0.0865	-0.0140	0.0644
h1	h09	0.0618	0.0865	-0.0140	0.0644
cx	c05	-0.1556	-0.0755	-0.0100	-0.1701
hc	h10	0.0872	0.1243	0.0357	0.0865
cx	c06	-0.1124	-0.2558	-0.1442	-0.1179
hc	h11	0.0783	0.1229	0.0689	0.0793
hc	h12	0.0783	0.1229	0.0689	0.0793
cx	c07	-0.1255	-0.2558	-0.1442	-0.1179
hc	h13	0.0742	0.1229	0.0689	0.0793
hc	h14	0.0742	0.1229	0.0689	0.0793
ca	c08	0.0216	-0.1043	-0.1262	0.0416
h5	h15	0.1794	0.2114	0.1823	0.1705
ca	c09	0.0235	-0.1043	-0.1262	0.0416
h5	h16	0.1793	0.2114	0.1823	0.1705
nh	n10	-0.7029	-0.0182	0.0884	-0.8384
ca	c11	0.3389	0.0476	-0.1071	0.4754
ca	c12	-0.2225	-0.0109	0.0325	-0.2349
no	n13	0.5848	0.6077	0.6193	0.6988
o	o14	-0.3355	-0.4049	-0.4151	-0.3826
o	o15	-0.3224	-0.4049	-0.4151	-0.3826
ca	c16	-0.2319	-0.0779	-0.0939	-0.2215
c3	c17	0.7177	0.5791	0.7361	0.3351
f	f01	-0.2258	-0.2069	-0.2504	-0.1061
f	f02	-0.2243	-0.2069	-0.2504	-0.1061
f	f03	-0.2247	-0.2069	-0.2504	-0.1061
ca	c18	-0.2326	-0.0109	0.0325	-0.2349
no	n19	0.5842	0.6077	0.6193	0.6988
o	o20	-0.3287	-0.4049	-0.4151	-0.3826
o	o21	-0.3339	-0.4049	-0.4151	-0.3826

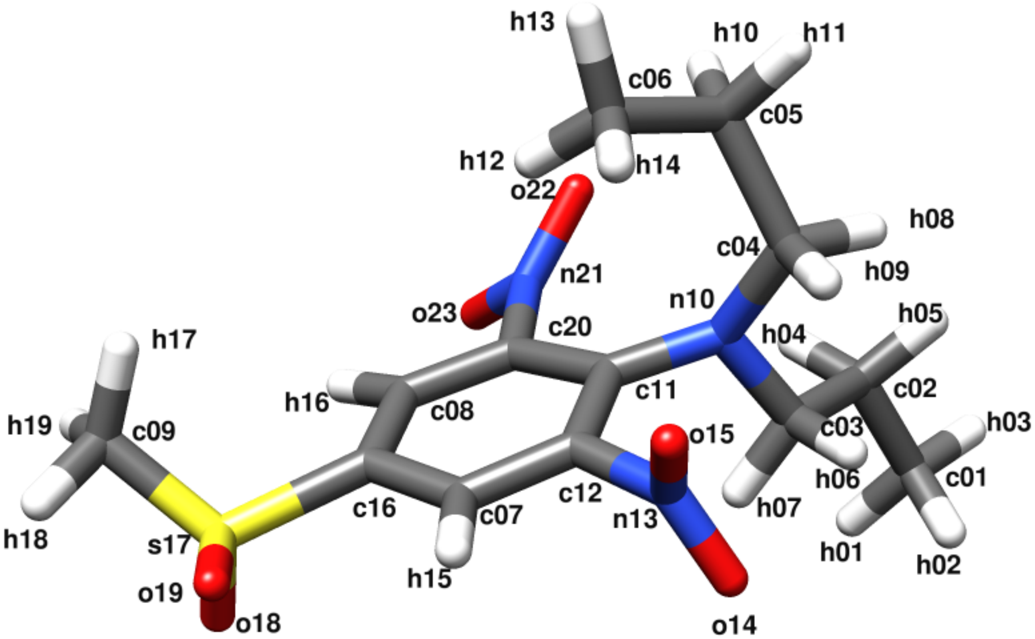

Figure S7: Atoms labels for nitralin.

Table S7: Atomic charges (e) for nitralin. For atoms labels see Figure S7.

Atom type	Atom label	AM1/BCC	RESP/HF/6-31G*	RESP-QM/MM	ABCG2
c3	c01	-0.0934	-0.1398	0.0240	-0.0980
hc	h01	0.0419	0.0368	-0.0103	0.0411
hc	h02	0.0419	0.0368	-0.0103	0.0411
hc	h03	0.0419	0.0368	-0.0103	0.0411
c3	c02	-0.0932	0.0847	0.1658	-0.0890
hc	h04	0.0536	0.0024	-0.0475	0.0538
hc	h05	0.0536	0.0024	-0.0475	0.0538
c3	c03	0.2062	-0.0441	0.1515	0.2013
h1	h06	0.0631	0.0677	-0.0067	0.0583
h1	h07	0.0631	0.0677	-0.0067	0.0583
c3	c04	0.2013	-0.0441	0.1515	0.2013
h1	h08	0.0640	0.0677	-0.0067	0.0583
h1	h09	0.0640	0.0677	-0.0067	0.0583
c3	c05	-0.0858	0.0847	0.1658	-0.0890
hc	h10	0.0511	0.0024	-0.0475	0.0538
hc	h11	0.0511	0.0024	-0.0475	0.0538
c3	c06	-0.0961	-0.1398	0.0240	-0.0980
hc	h12	0.0442	0.0368	-0.0103	0.0411
hc	h13	0.0442	0.0368	-0.0103	0.0411
hc	h14	0.0442	0.0368	-0.0103	0.0411
ca	c07	0.0786	-0.0960	-0.1257	0.0888
h5	h15	0.1902	0.1996	0.1628	0.1745
ca	c08	0.0680	-0.0960	-0.1257	0.0888
h5	h16	0.1735	0.1996	0.1628	0.1745
c3	c09	-0.2384	-0.3017	0.0058	-0.4825
h2	h17	0.1084	0.1235	0.0472	0.1221
h2	h18	0.1084	0.1235	0.0472	0.1221
h2	h19	0.1084	0.1235	0.0472	0.1221
nh	n10	-0.6717	-0.0713	-0.0361	-0.8238
ca	c11	0.3611	0.0667	-0.0599	0.4678
ca	c12	-0.2535	-0.0193	0.0309	-0.2377
no	n13	0.5822	0.6328	0.6197	0.7004
o	o14	-0.3320	-0.3994	-0.3926	-0.3806
o	o15	-0.3313	-0.3994	-0.3926	-0.3806
ca	c16	-0.3297	-0.1226	-0.0984	-0.4036
s6	s17	1.1388	1.0554	0.9338	1.3716
o	o18	-0.5868	-0.5682	-0.5479	-0.5745
o	o19	-0.5938	-0.5682	-0.5479	-0.5745
ca	c20	-0.2500	-0.0193	0.0309	-0.2377
no	n21	0.5798	0.6328	0.6197	0.7004
o	o22	-0.3282	-0.3994	-0.3926	-0.3806
o	o23	-0.3425	-0.3994	-0.3926	-0.3806

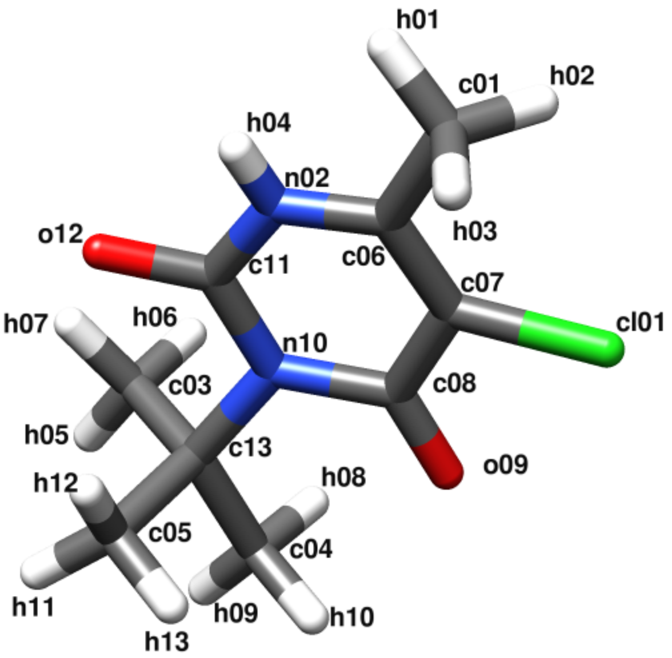

Figure S8: Atoms labels for terbacil.

Table S8: Atomic charges (e) for terbacil. For atom labels, see Figure S8.

Atom type	Atom label	AM1/BCC	RESP/HF/6-31G*	RESP-QM/MM	ABCG2
c3	c01	-0.0833	-0.2205	0.0377	-0.0775
h1	h01	0.0713	0.0975	0.0418	0.0704
h1	h02	0.0713	0.0975	0.0418	0.0704
h1	h03	0.0713	0.0975	0.0418	0.0704
n	n02	-0.2952	-0.4707	-0.3549	-0.5168
hn	h04	0.3168	0.3415	0.3507	0.4670
c3	c03	-0.0912	-0.2532	0.0537	-0.1070
hc	h05	0.0520	0.0648	-0.0512	0.0519
hc	h06	0.0520	0.0648	-0.0512	0.0519
hc	h07	0.0520	0.0648	-0.0512	0.0519
c3	c04	-0.1154	-0.2532	0.0537	-0.1070
hc	h08	0.0514	0.0648	-0.0512	0.0519
hc	h09	0.0514	0.0648	-0.0512	0.0519
hc	h10	0.0514	0.0648	-0.0512	0.0519
c3	c05	-0.1147	-0.2532	0.0537	-0.1070
hc	h11	0.0518	0.0648	-0.0512	0.0519
hc	h12	0.0518	0.0648	-0.0512	0.0519
hc	h13	0.0518	0.0648	-0.0512	0.0519
cc	c06	0.0763	0.2912	0.1769	0.1082
cc	c07	-0.1856	-0.2268	-0.2011	-0.1194
cl	cl01	-0.0430	-0.0901	-0.0805	-0.1196
c	c08	0.6182	0.5643	0.5230	0.5864
o	o09	-0.5899	-0.4807	-0.5518	-0.5166
n	n10	-0.1971	-0.3001	-0.3475	-0.3422
c	c11	0.5892	0.5494	0.4650	0.6020
o	o12	-0.6437	-0.5038	-0.4786	-0.5652
c3	c13	0.0792	0.4302	0.6354	0.1364

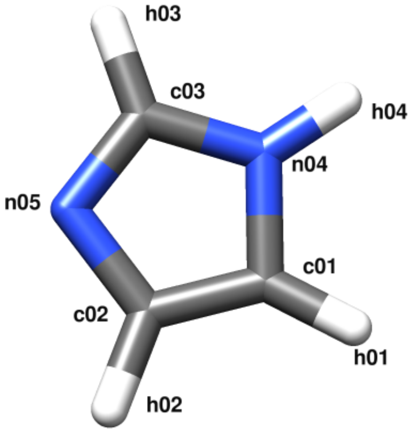

Figure S9: Atoms labels for imidazole.

Table S9: Atomic charges (e) for imidazole. For atoms labels see Figure S9.

Atom type	Atom label	AM1/BCC	RESP/HF/6-31G*	RESP-QM/MM	ABCG2
ca	c01	-0.2614	-0.2777	-0.246328	-0.2290
ha	h01	0.1761	0.1964	0.178507	0.1580
ca	c02	0.1165	0.1220	0.18158	0.2259
ha	h02	0.0422	0.1097	0.056156	0.0421
ca	c03	0.2073	0.1923	0.249869	0.3824
h4	h03	0.0598	0.1228	0.0706098	0.0601
nb	n04	-0.3221	-0.2695	-0.179325	-0.3215
hn	h04	0.2993	0.3226	0.331949	0.3500
nb	n05	-0.3175	-0.5185	-0.643018	-0.6680

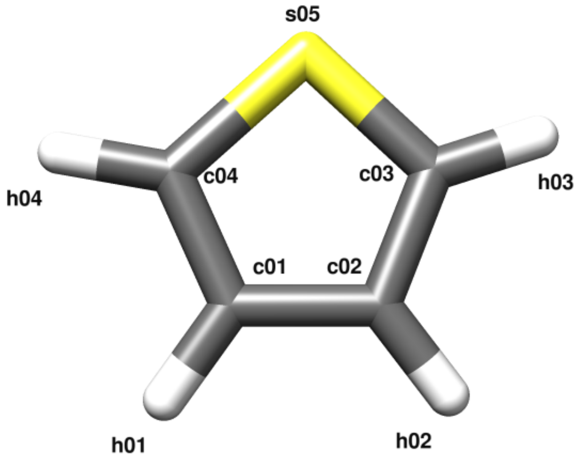

Figure S10: Atoms labels for thiophene.

Table S10: Atomic charges (e) for thiophene. For atom labels, see Figure S10.

Atom type	Atom label	AM1/BCC	RESP/HF/6-31G*	RESP-QM/MM	ABCG2
cd	c01	-0.1567	-0.14595	-0.12871	-0.1390
hc	h01	0.1524	0.14899	0.11221	0.1350
cc	c02	-0.1567	-0.14595	-0.12871	-0.1390
hc	h02	0.1524	0.14899	0.11221	0.1350
cd	c03	-0.1639	-0.20291	-0.13985	-0.2960
hc	h03	0.0767	0.20039	0.14887	0.1510
cc	c04	-0.1639	-0.20291	-0.13985	-0.2960
hc	h04	0.0767	0.20039	0.14887	0.1510
ss	s05	0.1831	-0.00103	0.01496	0.2980

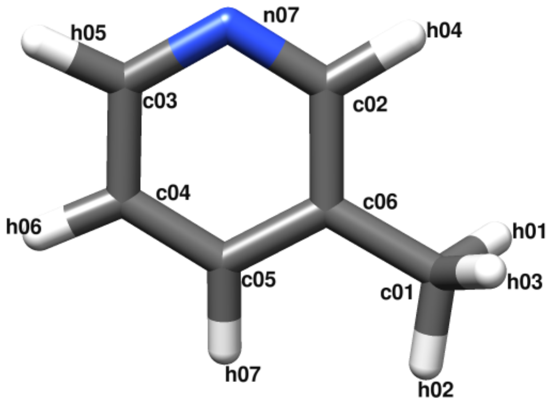

Figure S11: Atoms labels for 3-methyl-pyridine.

Table S11: Atomic charges (e) for 3-methyl-pyridine. For atoms labels see Figure S11.

Atom type	Atom label	AM1/BCC	RESP/HF/6-31G*	RESP-QM/MM	ABCG2
c3	c01	-0.0503	-0.3113	0.0771	-0.0508
hc	h01	0.0470	0.0974	0.0100	0.0470
hc	h02	0.0470	0.0940	0.0100	0.0470
hc	h03	0.0470	0.0945	0.0100	0.0470
ca	c02	0.2182	0.2137	0.3166	0.3267
h4	h04	0.0211	0.0786	0.0175	0.0211
ca	c03	0.2129	0.2461	0.3430	0.3217
h4	h05	0.0211	0.0845	0.0086	0.0211
ca	c04	-0.2422	-0.3066	-0.3014	-0.1590
ha	h06	0.1422	0.1537	0.1386	0.1240
ca	c05	-0.0969	-0.0760	0.0067	-0.0778
ha	h07	0.1372	0.1407	0.1150	0.1190
ca	c06	-0.1913	0.0448	-0.0970	-0.1263
nb	n07	-0.3130	-0.5541	-0.6547	-0.6607

Table S12: **RESP-QM/MM** atomic charges (e) of the 10 frames extracted from the QM/MM MD simulation of acetylsalicylic acid. For atom labels, see Figure S1.

A.t.	A.l.	1	2	3	4	5	6	7	8	9	10
c3	c01	-0.0819	-0.1894	-0.0583	-0.2199	-0.1130	-0.2510	-0.1026	0.0897	-0.1827	-0.2318
h1	h01	0.0581	0.0814	0.0422	0.0866	0.0544	0.0977	0.0635	0.0028	0.0727	0.0952
h1	h02	0.0581	0.0814	0.0422	0.0866	0.0544	0.0977	0.0635	0.0028	0.0727	0.0952
h1	h03	0.0581	0.0814	0.0422	0.0866	0.0544	0.0977	0.0635	0.0028	0.0727	0.0952
ca	c02	-0.1359	-0.1524	-0.1454	-0.2073	-0.1569	-0.1899	-0.1991	-0.0400	-0.0353	-0.0242
ha	h04	0.1436	0.1543	0.1575	0.1653	0.1187	0.1407	0.1812	0.1445	0.0999	0.1428
ca	c03	-0.1534	-0.0992	-0.1795	-0.1528	-0.0949	-0.1603	-0.1242	-0.1759	-0.3075	-0.2115
h4	h05	0.2150	0.1277	0.1973	0.1485	0.1251	0.1843	0.1366	0.1583	0.2228	0.1588
ca	c04	-0.1297	-0.1081	-0.0577	-0.0595	-0.1059	-0.0734	-0.0507	-0.0301	0.0449	-0.0850
ha	h06	0.1423	0.1197	0.1226	0.1399	0.1284	0.1382	0.1294	0.1073	0.1009	0.1459
ca	c05	-0.0244	-0.1006	-0.2025	-0.1629	-0.1501	-0.2172	-0.1618	-0.2529	-0.2113	-0.1998
h5	h07	0.0867	0.1048	0.1561	0.1361	0.1321	0.1585	0.1207	0.1782	0.1265	0.1323
c	c06	0.6065	0.5884	0.6890	0.7349	0.7398	0.6294	0.6390	0.6765	0.6887	0.7399
o	o07	-0.5375	-0.4402	-0.6125	-0.5818	-0.6194	-0.5179	-0.5580	-0.6023	-0.5220	-0.5364
os	o08	-0.2551	-0.2967	-0.4362	-0.4748	-0.4367	-0.3194	-0.3700	-0.3896	-0.4190	-0.4390
ca	c09	0.2205	0.2337	0.3467	0.3930	0.3151	0.2210	0.2686	0.2209	0.1984	0.2585
ca	c10	-0.2160	-0.0731	-0.1297	-0.1580	-0.1308	-0.0043	-0.0486	-0.0891	-0.0236	-0.0898
c	c11	0.7568	0.5920	0.7210	0.7661	0.7938	0.6885	0.7269	0.7814	0.7177	0.6832
o	o12	-0.5677	-0.5060	-0.5800	-0.5522	-0.5735	-0.6250	-0.5611	-0.6506	-0.6091	-0.5805
oh	o13	-0.5888	-0.6071	-0.5489	-0.6209	-0.5873	-0.5121	-0.6685	-0.5662	-0.5203	-0.5526
ho	h08	0.3444	0.4080	0.4340	0.4463	0.4520	0.4168	0.4515	0.4314	0.4130	0.4034

Table S13: **ABCG2** atomic charges (e) of the 10 frames extracted from the QM/MM MD simulation of acetylsalycilic acid. For atom labels, see Figure S1.

A.t.	A.l.	1	2	3	4	5	6	7	8	9	10
c3	c01	-0.1511	-0.1511	-0.1541	-0.1541	-0.1541	-0.1491	-0.1541	-0.1511	-0.1511	-0.1511
h1	h01	0.0844	0.0844	0.0860	0.0860	0.0860	0.0844	0.0860	0.0844	0.0844	0.0844
h1	h02	0.0844	0.0844	0.0860	0.0860	0.0860	0.0844	0.0860	0.0844	0.0844	0.0844
h1	h03	0.0844	0.0844	0.0860	0.0860	0.0860	0.0844	0.0860	0.0844	0.0844	0.0844
ca	c02	-0.1280	-0.1280	-0.1370	-0.1370	-0.1370	-0.1190	-0.1370	-0.1280	-0.1280	-0.1280
ha	h04	0.1450	0.1450	0.1470	0.1470	0.1470	0.1370	0.1470	0.1450	0.1450	0.1450
ca	c03	-0.0670	-0.0670	-0.0630	-0.0630	-0.0630	-0.0720	-0.0630	-0.0670	-0.0670	-0.0670
h4	h05	0.1260	0.1260	0.1260	0.1260	0.1260	0.1250	0.1260	0.1260	0.1260	0.1260
ca	c04	-0.1300	-0.1300	-0.1330	-0.1330	-0.1330	-0.1210	-0.1330	-0.1300	-0.1300	-0.1300
ha	h06	0.1270	0.1270	0.1270	0.1270	0.1270	0.1260	0.1270	0.1270	0.1270	0.1270
ca	c05	-0.0420	-0.0420	-0.0380	-0.0380	-0.0380	-0.0760	-0.0380	-0.0420	-0.0420	-0.0420
h5	h07	0.1430	0.1430	0.1450	0.1450	0.1450	0.1240	0.1450	0.1430	0.1430	0.1430
c	c06	0.6021	0.6021	0.5941	0.5941	0.5941	0.6021	0.5941	0.6021	0.6021	0.6021
o	o07	-0.4560	-0.4560	-0.4580	-0.4580	-0.4580	-0.4730	-0.4580	-0.4560	-0.4560	-0.4560
os	o08	-0.4012	-0.4012	-0.3902	-0.3902	-0.3902	-0.3702	-0.3902	-0.4012	-0.4012	-0.4012
ca	c09	0.1421	0.1421	0.1461	0.1461	0.1461	0.1751	0.1461	0.1421	0.1421	0.1421
ca	c10	-0.1816	-0.1816	-0.1846	-0.1846	-0.1846	-0.1806	-0.1846	-0.1816	-0.1816	-0.1816
c	c11	0.6117	0.6117	0.6137	0.6137	0.6137	0.6107	0.6137	0.6117	0.6117	0.6117
o	o12	-0.4540	-0.4540	-0.4560	-0.4560	-0.4560	-0.4450	-0.4560	-0.4540	-0.4540	-0.4540
oh	o13	-0.6131	-0.6131	-0.6091	-0.6091	-0.6091	-0.6111	-0.6091	-0.6131	-0.6131	-0.6131
ho	h08	0.4730	0.4730	0.4660	0.4660	0.4660	0.4650	0.4660	0.4730	0.4730	0.4730

Table S14: **RESP-QM/MM** atomic charges (e) of the 10 frames extracted from the QM/MM MD simulation of cyclohexanamine. For atom labels, see Figure S2.

A.t.	A.l.	1	2	3	4	5	6	7	8	9	10
c3	c01	0.1199	0.0056	0.1807	0.0728	0.1542	0.1620	0.0516	0.0203	-0.1105	0.1256
hc	h01	-0.0310	-0.0048	-0.0692	-0.0397	-0.0463	-0.0275	-0.0231	-0.0265	0.0271	-0.0706
hc	h02	-0.0310	-0.0048	-0.0692	-0.0397	-0.0463	-0.0275	-0.0231	-0.0265	0.0271	-0.0706
c3	c02	-0.0167	0.0458	0.1190	0.1046	-0.0329	-0.0749	0.0557	0.0508	0.1096	0.0199
hc	h03	0.0107	-0.0487	-0.0798	-0.0336	-0.0141	-0.0300	-0.0584	-0.0415	-0.0342	-0.0277
hc	h04	-0.0153	-0.0081	-0.0342	-0.0399	-0.0109	-0.0278	-0.0026	-0.0024	-0.0115	-0.0005
c3	c03	-0.0243	0.0464	-0.0875	-0.0995	0.0133	0.2072	-0.1004	-0.0030	-0.0804	-0.0096
hc	h05	-0.0472	-0.0272	-0.0098	-0.0428	-0.0200	-0.0674	-0.0359	-0.0017	-0.0260	-0.0343
hc	h06	-0.0232	-0.0665	-0.0309	0.0104	-0.0296	-0.0809	0.0197	-0.0613	0.0074	-0.0097
c3	c04	0.6518	0.6311	0.5280	0.6280	0.6815	0.5729	0.6898	0.6516	0.4870	0.5655
hc	h07	-0.0963	-0.1287	-0.0449	-0.1144	-0.1613	-0.1677	-0.1102	-0.0992	-0.0340	-0.0807
c3	c05	-0.0243	0.0464	-0.0875	-0.0995	0.0133	0.2072	-0.1004	-0.0030	-0.0804	-0.0096
hc	h08	-0.0472	-0.0272	-0.0098	-0.0428	-0.0200	-0.0674	-0.0359	-0.0017	-0.0260	-0.0343
hc	h09	-0.0232	-0.0665	-0.0309	0.0104	-0.0296	-0.0809	0.0197	-0.0613	0.0074	-0.0097
c3	c06	-0.0167	0.0458	0.1190	0.1046	-0.0329	-0.0749	0.0557	0.0508	0.1096	0.0199
hc	h10	0.0107	-0.0487	-0.0798	-0.0336	-0.0141	-0.0300	-0.0584	-0.0415	-0.0342	-0.0277
hc	h11	-0.0153	-0.0081	-0.0342	-0.0399	-0.0109	-0.0278	-0.0026	-0.0024	-0.0115	-0.0005
n3	n07	-1.0835	-1.1275	-1.0412	-0.9014	-1.1288	-1.1141	-1.1005	-1.1247	-0.9861	-1.0670
hn	h12	0.3509	0.3730	0.3810	0.2980	0.3677	0.3748	0.3796	0.3616	0.3297	0.3609
hn	h13	0.3509	0.3730	0.3810	0.2980	0.3677	0.3748	0.3796	0.3616	0.3297	0.3609

Table S15: **ABCG2** atomic charges (e) of the 10 frames extracted from the QM/MM MD simulation of cyclohexanamine. For atom labels, see Figure S2.

A.t.	A.l.	1	2	3	4	5	6	7	8	9	10
c3	c01	-0.0784	-0.0784	-0.0784	-0.0784	-0.0784	-0.0784	-0.0784	-0.0784	-0.0774	-0.0774
hc	h01	0.0392	0.0392	0.0392	0.0392	0.0392	0.0392	0.0392	0.0392	0.0402	0.0402
hc	h02	0.0392	0.0392	0.0392	0.0392	0.0392	0.0392	0.0392	0.0392	0.0402	0.0402
c3	c02	-0.0744	-0.0744	-0.0744	-0.0744	-0.0744	-0.0744	-0.0744	-0.0744	-0.0764	-0.0764
hc	h03	0.0405	0.0405	0.0405	0.0405	0.0405	0.0405	0.0405	0.0405	0.0392	0.0392
hc	h04	0.0405	0.0405	0.0405	0.0405	0.0405	0.0405	0.0405	0.0405	0.0392	0.0392
c3	c03	-0.0939	-0.0939	-0.0939	-0.0939	-0.0939	-0.0939	-0.0939	-0.0939	-0.0744	-0.0744
hc	h05	0.0417	0.0417	0.0417	0.0417	0.0417	0.0417	0.0417	0.0417	0.0457	0.0457
hc	h06	0.0417	0.0417	0.0417	0.0417	0.0417	0.0417	0.0417	0.0417	0.0457	0.0457
c3	c04	0.1625	0.1625	0.1625	0.1625	0.1625	0.1625	0.1625	0.1625	0.1715	0.1715
hc	h07	0.0587	0.0587	0.0587	0.0587	0.0587	0.0587	0.0587	0.0587	0.0057	0.0057
c3	c05	-0.0939	-0.0939	-0.0939	-0.0939	-0.0939	-0.0939	-0.0939	-0.0939	-0.0744	-0.0744
hc	h08	0.0417	0.0417	0.0417	0.0417	0.0417	0.0417	0.0417	0.0417	0.0457	0.0457
hc	h09	0.0417	0.0417	0.0417	0.0417	0.0417	0.0417	0.0417	0.0417	0.0457	0.0457
c3	c06	-0.0744	-0.0744	-0.0744	-0.0744	-0.0744	-0.0744	-0.0744	-0.0744	-0.0764	-0.0764
hc	h10	0.0405	0.0405	0.0405	0.0405	0.0405	0.0405	0.0405	0.0405	0.0392	0.0392
hc	h11	0.0405	0.0405	0.0405	0.0405	0.0405	0.0405	0.0405	0.0405	0.0392	0.0392
n3	n07	-1.0122	-1.0122	-1.0122	-1.0122	-1.0122	-1.0122	-1.0122	-1.0122	-1.0242	-1.0242
hn	h12	0.3995	0.3995	0.3995	0.3995	0.3995	0.3995	0.3995	0.3995	0.4040	0.4040
hn	h13	0.3995	0.3995	0.3995	0.3995	0.3995	0.3995	0.3995	0.3995	0.4040	0.4040

Table S16: **RESP-QM/MM** atomic charges (e) of the 10 frames extracted from the QM/MM MD simulation of 2-propoxyethanol. For atom labels, see Figure S3.

A.t.	A.l.	1	2	3	4	5	6	7	8	9	10
c3	c01	-0.0841	-0.1127	-0.1120	-0.1026	-0.0812	0.0124	-0.1661	-0.1115	-0.1316	-0.1856
hc	h01	0.0287	0.0024	0.0127	0.0125	-0.0012	-0.0113	0.0615	0.0294	0.0405	0.0570
hc	h02	0.0287	0.0024	0.0127	0.0125	-0.0012	-0.0113	0.0615	0.0294	0.0405	0.0570
hc	h03	0.0287	0.0024	0.0127	0.0125	-0.0012	-0.0113	0.0615	0.0294	0.0405	0.0570
c3	c02	0.1607	0.1691	0.2723	0.2861	0.3567	0.2227	0.0207	0.0629	0.0642	0.1237
hc	h04	-0.0499	-0.0345	-0.0362	-0.0886	-0.0552	-0.0496	0.0559	0.0116	0.0126	-0.0370
hc	h05	-0.0499	-0.0345	-0.0362	-0.0886	-0.0552	-0.0496	0.0559	0.0116	0.0126	-0.0370
c3	c03	0.2873	0.4752	0.0691	0.2159	0.0051	0.1659	-0.0840	0.2335	0.1290	0.2614
h1	h06	-0.0367	-0.0966	0.0292	-0.0176	0.0209	0.0212	0.1000	0.0010	0.0290	-0.0270
h1	h07	-0.0367	-0.0966	0.0292	-0.0176	0.0209	0.0212	0.1000	0.0010	0.0290	-0.0270
c3	c04	0.1181	0.3193	0.4564	0.3215	0.2712	0.3268	-0.0498	-0.0411	0.2585	0.3564
hc	h08	0.0255	-0.0345	-0.1044	-0.0229	-0.0126	-0.0399	0.0899	0.0403	-0.0016	-0.0399
hc	h09	0.0255	-0.0345	-0.1044	-0.0229	-0.0126	-0.0399	0.0899	0.0403	-0.0016	-0.0399
c3	c05	0.2157	0.4142	0.4080	0.2176	0.3318	0.4602	0.2780	0.2685	0.2781	0.1956
h1	h10	0.0032	-0.0662	-0.0719	0.0116	-0.0208	-0.0680	0.0056	0.0156	-0.0351	0.0045
h1	h11	0.0032	-0.0662	-0.0719	0.0116	-0.0208	-0.0680	0.0056	0.0156	-0.0351	0.0045
os	o06	-0.4342	-0.5230	-0.4761	-0.4333	-0.4362	-0.5724	-0.3956	-0.3881	-0.4746	-0.4263
oh	o07	-0.6340	-0.6914	-0.6870	-0.7587	-0.7174	-0.7722	-0.6238	-0.6634	-0.6478	-0.6781
ho	h12	0.4002	0.4055	0.3979	0.4508	0.4091	0.4631	0.3333	0.4141	0.3931	0.3811

Table S17: **ABCG2** atomic charges (e) of the 10 frames extracted from the QM/MM MD simulation of 2-propoxyethanol. For atom labels, see Figure S3.

A.t.	A.l.	1	2	3	4	5	6	7	8	9	10
c3	c01	0.0387	0.0387	0.0322	0.0322	0.0322	0.0322	0.0382	0.0382	0.0382	0.0382
hc	h01	-0.0911	-0.0911	-0.0911	-0.0911	-0.0911	-0.0911	-0.0911	-0.0911	-0.0911	-0.0911
hc	h02	0.0357	0.0357	0.0377	0.0377	0.0377	0.0377	0.0377	0.0377	0.0377	0.0377
hc	h03	0.0357	0.0357	0.0377	0.0377	0.0377	0.0377	0.0377	0.0377	0.0377	0.0377
c3	c02	0.1546	0.1546	0.1476	0.1476	0.1476	0.1476	0.1556	0.1556	0.1556	0.1556
hc	h04	0.0357	0.0357	0.0377	0.0377	0.0377	0.0377	0.0377	0.0377	0.0377	0.0377
hc	h05	-0.1164	-0.1164	-0.1124	-0.1124	-0.1124	-0.1124	-0.1114	-0.1114	-0.1114	-0.1114
c3	c03	0.0527	0.0527	0.0582	0.0582	0.0582	0.0582	0.0522	0.0522	0.0522	0.0522
h1	h06	0.0482	0.0482	0.0437	0.0437	0.0437	0.0437	0.0442	0.0442	0.0442	0.0442
h1	h07	0.0482	0.0482	0.0437	0.0437	0.0437	0.0437	0.0442	0.0442	0.0442	0.0442
c3	c04	0.0527	0.0527	0.0582	0.0582	0.0582	0.0582	0.0522	0.0522	0.0522	0.0522
hc	h08	0.1546	0.1546	0.1526	0.1526	0.1526	0.1526	0.1536	0.1536	0.1536	0.1536
hc	h09	0.0482	0.0482	0.0482	0.0482	0.0482	0.0482	0.0482	0.0482	0.0482	0.0482
c3	c05	-0.4800	-0.4800	-0.4660	-0.4660	-0.4660	-0.4660	-0.4800	-0.4800	-0.4800	-0.4800
h1	h10	0.0482	0.0482	0.0482	0.0482	0.0482	0.0482	0.0482	0.0482	0.0482	0.0482
h1	h11	0.1076	0.1076	0.1126	0.1126	0.1126	0.1126	0.1076	0.1076	0.1076	0.1076
os	o06	-0.7230	-0.7230	-0.7170	-0.7170	-0.7170	-0.7170	-0.7230	-0.7230	-0.7230	-0.7230
oh	o07	0.5120	0.5120	0.4960	0.4960	0.4960	0.4960	0.5120	0.5120	0.5120	0.5120
ho	h12	0.0387	0.0387	0.0322	0.0322	0.0322	0.0322	0.0382	0.0382	0.0382	0.0382

Table S18: **RESP-QM/MM** atomic charges (e) of the 10 frames extracted from the QM/MM MD simulation of ketoprofen. For atom labels, see Figure S4.

A.t.	A.l.	1	2	3	4	5	6	7	8	9	10
c3	c01	-0.0042	0.0279	-0.0376	0.1117	0.1254	0.0591	0.0655	0.0947	0.0132	-0.0916
hc	h01	-0.0002	-0.0051	0.0165	-0.0278	-0.0352	-0.0243	-0.0200	-0.0261	-0.0113	0.0000
hc	h02	-0.0002	-0.0051	0.0165	-0.0278	-0.0352	-0.0243	-0.0200	-0.0261	-0.0113	0.0000
hc	h03	-0.0002	-0.0051	0.0165	-0.0278	-0.0352	-0.0243	-0.0200	-0.0261	-0.0113	0.0000
c3	c02	0.2010	0.4259	0.2151	0.4482	0.3117	0.3399	0.2832	0.4035	0.3840	0.4533
hc	h04	-0.0475	-0.1059	-0.0462	-0.1213	-0.0753	-0.0644	-0.0509	-0.0702	-0.0527	-0.0296
ca	c03	-0.1354	-0.1755	-0.2414	-0.0396	-0.2607	-0.2629	-0.2848	-0.3347	-0.1988	-0.2514
h4	h05	0.1390	0.1022	0.1053	0.0805	0.0963	0.1466	0.1454	0.1494	0.1263	0.1381
ca	c04	-0.0860	-0.0449	-0.2246	-0.0922	-0.0409	-0.0067	-0.0735	-0.1117	-0.1139	-0.1235
h4	h06	0.0596	0.0705	0.1411	0.0751	0.1090	0.1031	0.0703	0.0938	0.0949	0.0927
ca	c05	-0.0954	-0.1633	-0.0637	-0.1385	-0.2458	-0.1999	-0.1419	-0.0700	-0.1376	-0.1258
ha	h07	0.1108	0.1610	0.1293	0.1545	0.1612	0.1430	0.1349	0.1273	0.1490	0.1436
ca	c06	-0.2555	-0.1088	-0.2565	-0.0689	-0.0881	-0.1132	-0.1665	-0.2419	-0.1512	-0.1469
h4	h08	0.1811	0.1047	0.1437	0.1094	0.1194	0.1408	0.1477	0.1346	0.1117	0.0562
ca	c07	-0.1881	-0.1352	-0.1749	-0.1608	-0.0655	-0.0576	-0.1025	-0.1401	-0.1054	-0.1305
ha	h09	0.1493	0.1082	0.0953	0.1235	0.0854	0.1002	0.1182	0.0718	0.0904	0.1113
ca	c08	-0.1154	-0.0769	0.0290	-0.1109	-0.1085	-0.1609	-0.1366	-0.0569	-0.1028	-0.1265
ha	h10	0.1245	0.1123	0.1005	0.1315	0.1092	0.1233	0.1281	0.0984	0.0977	0.1286
ca	c09	-0.1244	-0.1381	-0.2263	-0.0930	-0.0679	-0.0500	-0.0715	-0.1573	-0.1312	-0.1102
ha	h11	0.1228	0.1303	0.1305	0.1307	0.1327	0.1240	0.1340	0.1647	0.1506	0.1168
ca	c10	-0.1154	-0.0769	0.0290	-0.1109	-0.1085	-0.1609	-0.1366	-0.0569	-0.1028	-0.1265
ha	h12	0.1245	0.1123	0.1005	0.1315	0.1092	0.1233	0.1281	0.0984	0.0977	0.1286
ca	c11	-0.1881	-0.1352	-0.1749	-0.1608	-0.0655	-0.0576	-0.1025	-0.1401	-0.1054	-0.1305
h4	h13	0.1493	0.1082	0.0953	0.1235	0.0854	0.1002	0.1182	0.0718	0.0904	0.1113
ca	c12	0.0558	-0.1365	0.1544	-0.1474	0.0579	-0.0106	0.0028	0.0564	-0.0275	-0.0479
ca	c13	0.0011	0.0249	0.0645	-0.0955	0.0304	0.0619	0.0139	0.1158	0.0488	0.1038
c	c14	0.4295	0.3633	0.4745	0.4764	0.3409	0.2230	0.3839	0.3711	0.4107	0.3802
o	o15	-0.5690	-0.4173	-0.4655	-0.5645	-0.4243	-0.4751	-0.5223	-0.4640	-0.4828	-0.4816
ca	c16	0.1613	0.0552	-0.0201	0.0652	-0.0408	0.0454	0.0913	0.0757	0.0803	0.0985
c	c17	0.5577	0.4982	0.6640	0.4677	0.4398	0.4997	0.5758	0.4485	0.4985	0.5877
o	o18	-0.5521	-0.5339	-0.6196	-0.5414	-0.5301	-0.5370	-0.5835	-0.5106	-0.5708	-0.5819
oh	o19	-0.5389	-0.5839	-0.6908	-0.5534	-0.5693	-0.5808	-0.5557	-0.5845	-0.5744	-0.6290
ho	h14	0.4485	0.4425	0.5205	0.4531	0.4828	0.4771	0.4475	0.4413	0.4471	0.4826

Table S19: **ABCG2** atomic charges (e) of the 10 frames extracted from the QM/MM MD simulation of ketoprofen. For atom labels, see Figure S4.

A.t.	A.l.	1	2	3	4	5	6	7	8	9	10
c3	c01	-0.0911	-0.0901	-0.0901	-0.0901	-0.0901	-0.0901	-0.0901	-0.0901	-0.0901	-0.0911
hc	h01	0.0530	0.0517	0.0517	0.0517	0.0517	0.0517	0.0517	0.0517	0.0517	0.0530
hc	h02	0.0530	0.0517	0.0517	0.0517	0.0517	0.0517	0.0517	0.0517	0.0517	0.0530
hc	h03	0.0530	0.0517	0.0517	0.0517	0.0517	0.0517	0.0517	0.0517	0.0517	0.0530
c3	c02	-0.0564	-0.0544	-0.0544	-0.0544	-0.0544	-0.0544	-0.0544	-0.0544	-0.0544	-0.0564
hc	h04	0.0917	0.0917	0.0917	0.0917	0.0917	0.0917	0.0917	0.0917	0.0917	0.0917
ca	c03	-0.0480	-0.0470	-0.0470	-0.0470	-0.0470	-0.0470	-0.0470	-0.0470	-0.0470	-0.0480
h4	h05	0.1350	0.1360	0.1360	0.1360	0.1360	0.1360	0.1360	0.1360	0.1360	0.1350
ca	c04	-0.0680	-0.0680	-0.0680	-0.0680	-0.0680	-0.0680	-0.0680	-0.0680	-0.0680	-0.0680
h4	h06	0.1270	0.1270	0.1270	0.1270	0.1270	0.1270	0.1270	0.1270	0.1270	0.1270
ca	c05	-0.1250	-0.1260	-0.1260	-0.1260	-0.1260	-0.1260	-0.1260	-0.1260	-0.1260	-0.1250
ha	h07	0.1230	0.1220	0.1220	0.1220	0.1220	0.1220	0.1220	0.1220	0.1220	0.1230
ca	c06	-0.0720	-0.0740	-0.0740	-0.0740	-0.0740	-0.0740	-0.0740	-0.0740	-0.0740	-0.0720
h4	h08	0.1410	0.1350	0.1350	0.1350	0.1350	0.1350	0.1350	0.1350	0.1350	0.1410
ca	c07	-0.0675	-0.0665	-0.0665	-0.0665	-0.0665	-0.0665	-0.0665	-0.0665	-0.0665	-0.0675
ha	h09	0.1285	0.1295	0.1295	0.1295	0.1295	0.1295	0.1295	0.1295	0.1295	0.1285
ca	c08	-0.1245	-0.1255	-0.1255	-0.1255	-0.1255	-0.1255	-0.1255	-0.1255	-0.1255	-0.1245
ha	h10	0.1195	0.1195	0.1195	0.1195	0.1195	0.1195	0.1195	0.1195	0.1195	0.1195
ca	c09	-0.0860	-0.0860	-0.0860	-0.0860	-0.0860	-0.0860	-0.0860	-0.0860	-0.0860	-0.0860
ha	h11	0.1170	0.1170	0.1170	0.1170	0.1170	0.1170	0.1170	0.1170	0.1170	0.1170
ca	c10	-0.1245	-0.1255	-0.1255	-0.1255	-0.1255	-0.1255	-0.1255	-0.1255	-0.1255	-0.1245
ha	h12	0.1195	0.1195	0.1195	0.1195	0.1195	0.1195	0.1195	0.1195	0.1195	0.1195
ca	c11	-0.0675	-0.0665	-0.0665	-0.0665	-0.0665	-0.0665	-0.0665	-0.0665	-0.0665	-0.0675
h4	h13	0.1285	0.1295	0.1295	0.1295	0.1295	0.1295	0.1295	0.1295	0.1295	0.1285
ca	c12	-0.1063	-0.1033	-0.1033	-0.1033	-0.1033	-0.1033	-0.1033	-0.1033	-0.1033	-0.1063
ca	c13	-0.1696	-0.1686	-0.1686	-0.1686	-0.1686	-0.1686	-0.1686	-0.1686	-0.1686	-0.1696
c	c14	0.5893	0.5893	0.5893	0.5893	0.5893	0.5893	0.5893	0.5893	0.5893	0.5893
o	o15	-0.5291	-0.5281	-0.5281	-0.5281	-0.5281	-0.5281	-0.5281	-0.5281	-0.5281	-0.5291
ca	c16	-0.1696	-0.1696	-0.1696	-0.1696	-0.1696	-0.1696	-0.1696	-0.1696	-0.1696	-0.1696
c	c17	0.6011	0.5981	0.5981	0.5981	0.5981	0.5981	0.5981	0.5981	0.5981	0.6011
o	o18	-0.5140	-0.5050	-0.5050	-0.5050	-0.5050	-0.5050	-0.5050	-0.5050	-0.5050	-0.5140
oh	o19	-0.6381	-0.6441	-0.6441	-0.6441	-0.6441	-0.6441	-0.6441	-0.6441	-0.6441	-0.6381
ho	h14	0.4780	0.4790	0.4790	0.4790	0.4790	0.4790	0.4790	0.4790	0.4790	0.4780

Table S20: **RESP-QM/MM** atomic charges (e) of the 10 frames extracted from the QM/MM MD simulation of dialifor. For atom labels, see Figure S5.

A.t.	A.l.	1	2	3	4	5	6	7	8	9	10
c3	c01	0.3381	0.3353	0.2552	0.2056	0.3005	-0.0123	0.1048	0.2451	0.1088	0.0454
hc	h01	-0.1057	-0.0773	-0.0786	-0.0629	-0.0709	-0.0068	-0.0307	-0.0558	-0.0257	-0.0221
hc	h02	-0.1057	-0.0773	-0.0786	-0.0629	-0.0709	-0.0068	-0.0307	-0.0558	-0.0257	-0.0221
hc	h03	-0.1057	-0.0773	-0.0786	-0.0629	-0.0709	-0.0068	-0.0307	-0.0558	-0.0257	-0.0221
c3	c02	0.5590	0.3567	0.6056	0.5781	0.4663	0.4389	0.4793	0.4020	0.5174	0.5238
h1	h04	-0.0978	-0.0874	-0.1411	-0.1350	-0.1172	-0.0189	-0.0818	-0.0987	-0.0980	-0.0888
h1	h05	-0.0978	-0.0874	-0.1411	-0.1350	-0.1172	-0.0189	-0.0818	-0.0987	-0.0980	-0.0888
c3	c03	0.5590	0.3567	0.6056	0.5781	0.4663	0.4389	0.4793	0.4020	0.5174	0.5238
h1	h06	-0.0978	-0.0874	-0.1411	-0.1350	-0.1172	-0.0189	-0.0818	-0.0987	-0.0980	-0.0888
h1	h07	-0.0978	-0.0874	-0.1411	-0.1350	-0.1172	-0.0189	-0.0818	-0.0987	-0.0980	-0.0888
c3	c04	0.3381	0.3353	0.2552	0.2056	0.3005	-0.0123	0.1048	0.2451	0.1088	0.0454
hc	h08	-0.1057	-0.0773	-0.0786	-0.0629	-0.0709	-0.0068	-0.0307	-0.0558	-0.0257	-0.0221
hc	h09	-0.1057	-0.0773	-0.0786	-0.0629	-0.0709	-0.0068	-0.0307	-0.0558	-0.0257	-0.0221
hc	h10	-0.1057	-0.0773	-0.0786	-0.0629	-0.0709	-0.0068	-0.0307	-0.0558	-0.0257	-0.0221
c3	c05	0.8316	0.6747	0.8523	0.8702	0.9052	0.7635	0.8152	0.7054	0.4753	0.8121
h2	h11	-0.1207	-0.1691	-0.0565	-0.0872	-0.0736	-0.1087	-0.0781	-0.0534	0.0418	-0.2106
c3	c06	0.1723	0.2734	0.1541	0.2489	0.0918	-0.0222	0.0622	-0.0914	0.0805	0.3463
hc	h12	-0.0242	-0.0444	-0.0195	-0.0234	-0.0064	0.0513	0.0255	0.0875	0.0479	-0.0478
hc	h13	-0.0242	-0.0444	-0.0195	-0.0234	-0.0064	0.0513	0.0255	0.0875	0.0479	-0.0478
cl	cl01	-0.2749	-0.2564	-0.2175	-0.3388	-0.2442	-0.1979	-0.2003	-0.2147	-0.2075	-0.2893
ca	c07	-0.0965	-0.1170	-0.0281	-0.0795	-0.1797	-0.1159	-0.0757	-0.0953	-0.0433	-0.0921
h5	h14	0.1289	0.1135	0.1050	0.1399	0.1406	0.1308	0.1353	0.1269	0.1110	0.1336
ca	c08	-0.1082	-0.0918	-0.1513	-0.0964	-0.0561	-0.0753	-0.1096	-0.0741	-0.1181	-0.1113
ha	h15	0.1601	0.1472	0.1694	0.1300	0.1311	0.1215	0.1300	0.1295	0.1416	0.1393
ca	c09	-0.1082	-0.0918	-0.1513	-0.0964	-0.0561	-0.0753	-0.1096	-0.0741	-0.1181	-0.1113
ha	h16	0.1601	0.1472	0.1694	0.1300	0.1311	0.1215	0.1300	0.1295	0.1416	0.1393
ca	c10	-0.0965	-0.1170	-0.0281	-0.0795	-0.1797	-0.1159	-0.0757	-0.0953	-0.0433	-0.0921
h5	h17	0.1289	0.1135	0.1050	0.1399	0.1406	0.1308	0.1353	0.1269	0.1110	0.1336
os	o11	-0.5100	-0.3441	-0.5021	-0.5353	-0.3933	-0.4082	-0.4401	-0.3990	-0.4580	-0.4178
p5	p12	0.5384	0.6376	0.8239	0.8755	0.7248	0.5129	0.7633	0.7787	0.7382	0.6403
s2	s13	-0.3556	-0.4449	-0.3642	-0.3414	-0.3354	-0.3490	-0.4486	-0.3195	-0.4446	-0.3542
os	o14	-0.5100	-0.3441	-0.5021	-0.5353	-0.3933	-0.4082	-0.4401	-0.3990	-0.4580	-0.4178
ss	s15	-0.2408	-0.3570	-0.4317	-0.4899	-0.5243	-0.3816	-0.4754	-0.4542	-0.4333	-0.4594
n	n16	-0.2027	0.0963	-0.4193	-0.1812	-0.1926	-0.0904	-0.1030	-0.2539	0.1671	0.1115
c	c17	0.2060	0.1150	0.4208	0.2664	0.2295	0.1829	0.2034	0.3257	0.1698	0.0597
c	c18	0.2060	0.1150	0.4208	0.2664	0.2295	0.1829	0.2034	0.3257	0.1698	0.0597
o	o19	-0.4326	-0.3764	-0.5000	-0.4429	-0.4613	-0.3989	-0.4314	-0.4600	-0.4427	-0.3697
o	o20	-0.4326	-0.3764	-0.5000	-0.4429	-0.4613	-0.3989	-0.4314	-0.4600	-0.4427	-0.3697
ca	c21	0.1184	0.0855	-0.0077	0.0382	0.1002	0.0802	0.0668	0.0279	0.0296	0.0824
ca	c22	0.1184	0.0855	-0.0077	0.0382	0.1002	0.0802	0.0668	0.0279	0.0296	0.0824

Table S21: **ABCG2** atomic charges (e) of the 10 frames extracted from the QM/MM MD simulation of dialifor. For atom labels, see Figure S5.

A.t.	A.l.	1	2	3	4	5	6	7	8	9	10
c3	c01	0.1137	0.1137	0.1142	0.1142	0.1097	0.1097	0.1097	0.1137	0.1112	0.1112
hc	h01	-0.1041	-0.1261	-0.1226	-0.1176	-0.1146	-0.1146	-0.1146	-0.1221	-0.1306	-0.1306
hc	h02	0.0547	0.0579	0.0560	0.0562	0.0550	0.0550	0.0552	0.0562	0.0612	0.0612
hc	h03	0.0547	0.0579	0.0560	0.0562	0.0550	0.0550	0.0552	0.0562	0.0612	0.0612
c3	c02	0.1137	0.1137	0.1142	0.1142	0.1097	0.1097	0.1097	0.1137	0.1112	0.1112
h1	h04	0.0547	0.0579	0.0560	0.0562	0.0550	0.0550	0.0552	0.0562	0.0612	0.0612
h1	h05	-0.0359	-0.0194	-0.0214	-0.0194	-0.0359	-0.0359	-0.0359	-0.0209	-0.0374	-0.0374
c3	c03	-0.2610	-0.2590	-0.2590	-0.2590	-0.2630	-0.2630	-0.2630	-0.2580	-0.2620	-0.2620
h1	h06	0.0672	0.0675	0.0682	0.0652	0.0787	0.0787	0.0787	0.0682	0.0780	0.0780
h1	h07	0.0672	0.0675	0.0682	0.0652	0.0787	0.0787	0.0787	0.0682	0.0780	0.0780
c3	c04	-0.0410	-0.0415	-0.0415	-0.0410	-0.0395	-0.0395	-0.0395	-0.0410	-0.0395	-0.0395
hc	h08	-0.0359	-0.0194	-0.0214	-0.0194	-0.0359	-0.0359	-0.0359	-0.0209	-0.0374	-0.0374
hc	h09	0.0672	0.0675	0.0682	0.0652	0.0787	0.0787	0.0787	0.0682	0.0780	0.0780
hc	h10	0.0672	0.0675	0.0682	0.0652	0.0787	0.0787	0.0787	0.0682	0.0780	0.0780
c3	c05	0.1445	0.1435	0.1440	0.1440	0.1435	0.1435	0.1435	0.1435	0.1440	0.1440
h2	h11	-0.1041	-0.1261	-0.1226	-0.1176	-0.1146	-0.1146	-0.1146	-0.1221	-0.1306	-0.1306
c3	c06	-0.0965	-0.0960	-0.0965	-0.0965	-0.0955	-0.0955	-0.0955	-0.0965	-0.0950	-0.0950
hc	h12	0.0547	0.0579	0.0560	0.0562	0.0550	0.0550	0.0552	0.0562	0.0612	0.0612
hc	h13	0.0547	0.0579	0.0560	0.0562	0.0550	0.0550	0.0552	0.0562	0.0612	0.0612
cl	cl01	-0.1346	-0.1376	-0.1371	-0.1371	-0.1346	-0.1346	-0.1346	-0.1346	-0.1346	-0.1346
ca	c07	-0.0410	-0.0415	-0.0415	-0.0410	-0.0395	-0.0395	-0.0395	-0.0410	-0.0395	-0.0395
h5	h14	0.0996	0.1016	0.1016	0.1036	0.0986	0.0986	0.0986	0.1016	0.0996	0.0996
ca	c08	0.1290	0.1295	0.1295	0.1295	0.1295	0.1295	0.1295	0.1285	0.1300	0.1300
ha	h15	0.1237	0.1207	0.1197	0.1167	0.1307	0.1307	0.1307	0.1137	0.1267	0.1267
ca	c09	-0.0965	-0.0960	-0.0965	-0.0965	-0.0955	-0.0955	-0.0955	-0.0965	-0.0950	-0.0950
ha	h16	0.2717	0.2737	0.2707	0.2697	0.2657	0.2657	0.2657	0.2787	0.2717	0.2717
ca	c10	0.1290	0.1295	0.1295	0.1295	0.1295	0.1295	0.1295	0.1285	0.1300	0.1300
h5	h17	0.0547	0.0579	0.0560	0.0562	0.0550	0.0550	0.0552	0.0562	0.0612	0.0612
os	o11	-0.4124	-0.4084	-0.3994	-0.4014	-0.4394	-0.4394	-0.4394	-0.4134	-0.4404	-0.4404
p5	p12	-0.4710	-0.4740	-0.4735	-0.4765	-0.4800	-0.4800	-0.4800	-0.4750	-0.4790	-0.4790
s2	s13	-0.4710	-0.4740	-0.4735	-0.4765	-0.4800	-0.4800	-0.4800	-0.4750	-0.4790	-0.4790
os	o14	-0.3964	-0.3874	-0.3884	-0.3854	-0.3984	-0.3984	-0.3984	-0.3854	-0.3984	-0.3984
ss	s15	-0.1346	-0.1376	-0.1371	-0.1371	-0.1346	-0.1346	-0.1346	-0.1346	-0.1346	-0.1346
n	n16	-0.3239	-0.3344	-0.3369	-0.3394	-0.3179	-0.3179	-0.3179	-0.3379	-0.3189	-0.3189
c	c17	-0.3239	-0.3344	-0.3369	-0.3394	-0.3179	-0.3179	-0.3179	-0.3379	-0.3189	-0.3189
c	c18	0.1445	0.1435	0.1440	0.1440	0.1435	0.1435	0.1435	0.1435	0.1440	0.1440
o	o19	0.5876	0.5866	0.5871	0.5886	0.5896	0.5896	0.5896	0.5906	0.5906	0.5906
o	o20	0.5876	0.5866	0.5871	0.5886	0.5896	0.5896	0.5896	0.5906	0.5906	0.5906
ca	c21	-0.4650	-0.4620	-0.4620	-0.4590	-0.4670	-0.4670	-0.4670	-0.4650	-0.4730	-0.4730
ca	c22	0.9072	0.9132	0.9162	0.9192	0.9192	0.9192	0.9192	0.9222	0.9162	0.9162

Table S22: **RESP-QM/MM** atomic charges (e) of the 10 frames extracted from the QM/MM MD simulation of profluralin. For atom labels, see Figure S6.

A.t.	A.l.	1	2	3	4	5	6	7	8	9	10
c3	c01	-0.1475	-0.0557	0.0033	0.1592	-0.1595	0.1411	-0.0535	-0.0444	0.0342	-0.0526
hc	h01	0.0352	0.0188	-0.0033	-0.0295	0.0356	-0.0373	0.0026	0.0076	-0.0034	0.0062
hc	h02	0.0352	0.0188	-0.0033	-0.0295	0.0356	-0.0373	0.0026	0.0076	-0.0034	0.0062
hc	h03	0.0352	0.0188	-0.0033	-0.0295	0.0356	-0.0373	0.0026	0.0076	-0.0034	0.0062
c3	c02	0.2560	0.1797	0.1182	0.0840	0.5000	0.0771	0.2367	0.2130	-0.0169	0.1828
hc	h04	-0.0856	-0.0521	-0.0245	-0.0408	-0.1299	-0.0377	-0.0500	-0.0782	0.0072	-0.0493
hc	h05	-0.0856	-0.0521	-0.0245	-0.0408	-0.1299	-0.0377	-0.0500	-0.0782	0.0072	-0.0493
c3	c03	0.2682	0.1254	0.1866	-0.0362	-0.0981	0.3337	0.1623	0.0506	0.2212	0.1106
h1	h06	-0.0439	-0.0155	-0.0306	0.0541	-0.0305	-0.0481	-0.0095	0.0193	-0.0074	-0.0256
h1	h07	-0.0439	-0.0155	-0.0306	0.0541	-0.0305	-0.0481	-0.0095	0.0193	-0.0074	-0.0256
c3	c04	0.0850	0.0328	0.1054	0.3751	0.2585	0.2067	0.2100	0.3534	0.2756	0.2488
h1	h08	0.0154	0.0265	0.0078	-0.0455	-0.0190	-0.0105	-0.0012	-0.0379	-0.0327	-0.0432
h1	h09	0.0154	0.0265	0.0078	-0.0455	-0.0190	-0.0105	-0.0012	-0.0379	-0.0327	-0.0432
cx	c05	-0.0356	0.1060	0.0405	-0.2234	-0.1024	0.1439	-0.0354	0.1598	-0.1021	-0.0516
hc	h10	0.0080	0.0530	0.0065	0.1078	0.0690	-0.0669	0.0440	0.0131	0.0770	0.0454
cx	c06	-0.1388	-0.1332	-0.1338	-0.1400	-0.1038	-0.1917	-0.1111	-0.2750	-0.0807	-0.1338
hc	h11	0.0777	0.0412	0.0685	0.0863	0.0440	0.0927	0.0606	0.1027	0.0501	0.0655
hc	h12	0.0777	0.0412	0.0685	0.0863	0.0440	0.0927	0.0606	0.1027	0.0501	0.0655
cx	c07	-0.1388	-0.1332	-0.1338	-0.1400	-0.1038	-0.1917	-0.1111	-0.2750	-0.0807	-0.1338
hc	h13	0.0777	0.0412	0.0685	0.0863	0.0440	0.0927	0.0606	0.1027	0.0501	0.0655
hc	h14	0.0777	0.0412	0.0685	0.0863	0.0440	0.0927	0.0606	0.1027	0.0501	0.0655
ca	c08	-0.1406	-0.2035	-0.1531	-0.1235	-0.1921	-0.0048	-0.1337	-0.1649	-0.0535	-0.0921
h5	h15	0.1697	0.2115	0.1764	0.1750	0.1938	0.1649	0.1837	0.2024	0.1630	0.1830
ca	c09	-0.1406	-0.2035	-0.1531	-0.1235	-0.1921	-0.0048	-0.1337	-0.1649	-0.0535	-0.0921
h5	h16	0.1697	0.2115	0.1764	0.1750	0.1938	0.1649	0.1837	0.2024	0.1630	0.1830
nh	n10	-0.0329	0.1759	0.0767	0.2540	0.1981	-0.0846	-0.0114	0.0910	-0.0591	0.2766
ca	c11	0.0996	-0.1828	-0.1662	-0.1911	-0.1296	-0.0232	-0.0116	-0.2619	-0.0105	-0.1940
ca	c12	-0.0995	0.1380	0.0682	0.0187	0.0239	0.0711	-0.0182	0.0946	-0.0291	0.0574
no	n13	0.6774	0.5313	0.6541	0.6477	0.7079	0.5363	0.6411	0.5985	0.6588	0.5400
o	o14	-0.3944	-0.3986	-0.4270	-0.4391	-0.4245	-0.3987	-0.4221	-0.4193	-0.4261	-0.4012
o	o15	-0.3944	-0.3986	-0.4270	-0.4391	-0.4245	-0.3987	-0.4221	-0.4193	-0.4261	-0.4012
ca	c16	-0.0533	-0.0346	-0.0378	-0.0682	0.0132	-0.3928	-0.0560	-0.0271	-0.1790	-0.1039
c3	c17	0.7801	0.6867	0.7107	0.7535	0.7094	0.8878	0.6581	0.7129	0.7660	0.6959
f	f01	-0.2582	-0.2398	-0.2431	-0.2688	-0.2481	-0.2819	-0.2358	-0.2449	-0.2477	-0.2357
f	f02	-0.2582	-0.2398	-0.2431	-0.2688	-0.2481	-0.2819	-0.2358	-0.2449	-0.2477	-0.2357
f	f03	-0.2582	-0.2398	-0.2431	-0.2688	-0.2481	-0.2819	-0.2358	-0.2449	-0.2477	-0.2357
ca	c18	-0.0995	0.1380	0.0682	0.0187	0.0239	0.0711	-0.0182	0.0946	-0.0291	0.0574
no	n19	0.6774	0.5313	0.6541	0.6477	0.7079	0.5363	0.6411	0.5985	0.6588	0.5400
o	o20	-0.3944	-0.3986	-0.4270	-0.4391	-0.4245	-0.3987	-0.4221	-0.4193	-0.4261	-0.4012
o	o21	-0.3944	-0.3986	-0.4270	-0.4391	-0.4245	-0.3987	-0.4221	-0.4193	-0.4261	-0.4012

Table S23: **ABCG2** atomic charges (e) of the 10 frames extracted from the QM/MM MD simulation of profluralin. For atom labels, see Figure S6.

A.t.	A.l.	1	2	3	4	5	6	7	8	9	10
c3	c01	-0.0951	-0.0911	-0.0951	-0.0951	-0.0951	-0.0911	-0.0951	-0.0951	-0.0951	-0.0951
hc	h01	0.0400	0.0387	0.0400	0.0400	0.0400	0.0387	0.0400	0.0400	0.0400	0.0400
hc	h02	0.0400	0.0387	0.0400	0.0400	0.0400	0.0387	0.0400	0.0400	0.0400	0.0400
hc	h03	0.0400	0.0387	0.0400	0.0400	0.0400	0.0387	0.0400	0.0400	0.0400	0.0400
c3	c02	-0.1004	-0.1124	-0.1004	-0.1004	-0.1004	-0.1124	-0.1004	-0.1004	-0.1004	-0.1004
hc	h04	0.0567	0.0572	0.0572	0.0567	0.0567	0.0572	0.0567	0.0567	0.0567	0.0567
hc	h05	0.0567	0.0572	0.0572	0.0567	0.0567	0.0572	0.0567	0.0567	0.0567	0.0567
c3	c03	0.2188	0.2128	0.2188	0.2188	0.2188	0.2128	0.2188	0.2188	0.2188	0.2188
h1	h06	0.0587	0.0682	0.0587	0.0587	0.0587	0.0682	0.0587	0.0587	0.0587	0.0587
h1	h07	0.0587	0.0682	0.0587	0.0587	0.0587	0.0682	0.0587	0.0587	0.0587	0.0587
c3	c04	0.2448	0.2258	0.2448	0.2448	0.2448	0.2258	0.2448	0.2448	0.2448	0.2448
h1	h08	0.0652	0.0617	0.0647	0.0647	0.0652	0.0617	0.0652	0.0652	0.0652	0.0652
h1	h09	0.0652	0.0617	0.0647	0.0647	0.0652	0.0617	0.0652	0.0652	0.0652	0.0652
cx	c05	-0.1727	-0.1607	-0.1717	-0.1717	-0.1727	-0.1607	-0.1727	-0.1727	-0.1727	-0.1727
hc	h10	0.0877	0.0817	0.0877	0.0877	0.0877	0.0817	0.0877	0.0877	0.0877	0.0877
cx	c06	-0.1179	-0.1179	-0.1179	-0.1179	-0.1179	-0.1179	-0.1179	-0.1179	-0.1179	-0.1179
hc	h11	0.0797	0.0777	0.0797	0.0797	0.0797	0.0777	0.0797	0.0797	0.0797	0.0797
hc	h12	0.0797	0.0777	0.0797	0.0797	0.0797	0.0777	0.0797	0.0797	0.0797	0.0797
cx	c07	-0.1179	-0.1179	-0.1179	-0.1179	-0.1179	-0.1179	-0.1179	-0.1179	-0.1179	-0.1179
hc	h13	0.0797	0.0777	0.0797	0.0797	0.0797	0.0777	0.0797	0.0797	0.0797	0.0797
hc	h14	0.0797	0.0777	0.0797	0.0797	0.0797	0.0777	0.0797	0.0797	0.0797	0.0797
ca	c08	0.0445	0.0300	0.0445	0.0445	0.0445	0.0300	0.0445	0.0445	0.0445	0.0445
h5	h15	0.1705	0.1700	0.1710	0.1710	0.1705	0.1700	0.1705	0.1705	0.1705	0.1705
ca	c09	0.0445	0.0300	0.0445	0.0445	0.0445	0.0300	0.0445	0.0445	0.0445	0.0445
h5	h16	0.1705	0.1700	0.1710	0.1710	0.1705	0.1700	0.1705	0.1705	0.1705	0.1705
nh	n10	-0.8384	-0.8384	-0.8384	-0.8384	-0.8384	-0.8384	-0.8384	-0.8384	-0.8384	-0.8384
ca	c11	0.4810	0.4530	0.4810	0.4810	0.4810	0.4530	0.4810	0.4810	0.4810	0.4810
ca	c12	-0.2397	-0.2157	-0.2397	-0.2397	-0.2397	-0.2157	-0.2397	-0.2397	-0.2397	-0.2397
no	n13	0.6982	0.7012	0.6982	0.6982	0.6982	0.7012	0.6982	0.6982	0.6982	0.6982
o	o14	-0.3833	-0.3800	-0.3833	-0.3833	-0.3833	-0.3800	-0.3833	-0.3833	-0.3833	-0.3833
o	o15	-0.3833	-0.3800	-0.3833	-0.3833	-0.3833	-0.3800	-0.3833	-0.3833	-0.3833	-0.3833
ca	c16	-0.2253	-0.2063	-0.2253	-0.2253	-0.2253	-0.2063	-0.2253	-0.2253	-0.2253	-0.2253
c3	c17	0.3353	0.3343	0.3353	0.3353	0.3353	0.3343	0.3353	0.3353	0.3353	0.3353
f	f01	-0.1063	-0.1053	-0.1063	-0.1063	-0.1063	-0.1053	-0.1063	-0.1063	-0.1063	-0.1063
f	f02	-0.1063	-0.1053	-0.1063	-0.1063	-0.1063	-0.1053	-0.1063	-0.1063	-0.1063	-0.1063
f	f03	-0.1063	-0.1053	-0.1063	-0.1063	-0.1063	-0.1053	-0.1063	-0.1063	-0.1063	-0.1063
ca	c18	-0.2397	-0.2157	-0.2397	-0.2397	-0.2397	-0.2157	-0.2397	-0.2397	-0.2397	-0.2397
no	n19	0.6982	0.7012	0.6982	0.6982	0.6982	0.7012	0.6982	0.6982	0.6982	0.6982
o	o20	-0.3833	-0.3800	-0.3833	-0.3833	-0.3833	-0.3800	-0.3833	-0.3833	-0.3833	-0.3833
o	o21	-0.3833	-0.3800	-0.3833	-0.3833	-0.3833	-0.3800	-0.3833	-0.3833	-0.3833	-0.3833

Table S24: **RESP-QM/MM** atomic charges (e) of the 10 frames extracted from the QM/MM MD simulation of nitralin. For atom labels, see Figure S7.

A.t.	A.l.	1	2	3	4	5	6	7	8	9	10
c3	c01	0.0439	-0.0420	0.0147	0.0263	0.1737	-0.0672	0.0887	0.0684	-0.0726	0.0068
hc	h01	-0.0141	0.0071	-0.0174	-0.0099	-0.0458	0.0087	-0.0279	-0.0194	0.0151	0.0002
hc	h02	-0.0141	0.0071	-0.0174	-0.0099	-0.0458	0.0087	-0.0279	-0.0194	0.0151	0.0002
hc	h03	-0.0141	0.0071	-0.0174	-0.0099	-0.0458	0.0087	-0.0279	-0.0194	0.0151	0.0002
c3	c02	0.0180	0.0988	0.3656	0.0981	0.2312	0.2485	-0.0222	0.0994	0.2456	0.2751
hc	h04	-0.0105	-0.0368	-0.0983	-0.0194	-0.0669	-0.0677	0.0177	-0.0366	-0.0532	-0.1032
hc	h05	-0.0105	-0.0368	-0.0983	-0.0194	-0.0669	-0.0677	0.0177	-0.0366	-0.0532	-0.1032
c3	c03	0.2525	0.2364	0.1966	0.1031	0.1316	0.2472	0.1555	0.2292	-0.0781	0.0410
h1	h06	-0.0098	-0.0462	-0.0501	-0.0059	-0.0041	-0.0261	0.0139	-0.0075	0.0486	0.0201
h1	h07	-0.0098	-0.0462	-0.0501	-0.0059	-0.0041	-0.0261	0.0139	-0.0075	0.0486	0.0201
c3	c04	0.2525	0.2364	0.1966	0.1031	0.1316	0.2472	0.1555	0.2292	-0.0781	0.0410
h1	h08	-0.0098	-0.0462	-0.0501	-0.0059	-0.0041	-0.0261	0.0139	-0.0075	0.0486	0.0201
h1	h09	-0.0098	-0.0462	-0.0501	-0.0059	-0.0041	-0.0261	0.0139	-0.0075	0.0486	0.0201
c3	c05	0.0180	0.0988	0.3656	0.0981	0.2312	0.2485	-0.0222	0.0994	0.2456	0.2751
hc	h10	-0.0105	-0.0368	-0.0983	-0.0194	-0.0669	-0.0677	0.0177	-0.0366	-0.0532	-0.1032
hc	h11	-0.0105	-0.0368	-0.0983	-0.0194	-0.0669	-0.0677	0.0177	-0.0366	-0.0532	-0.1032
c3	c06	0.0439	-0.0420	0.0147	0.0263	0.1737	-0.0672	0.0887	0.0684	-0.0726	0.0068
hc	h12	-0.0141	0.0071	-0.0174	-0.0099	-0.0458	0.0087	-0.0279	-0.0194	0.0151	0.0002
hc	h13	-0.0141	0.0071	-0.0174	-0.0099	-0.0458	0.0087	-0.0279	-0.0194	0.0151	0.0002
hc	h14	-0.0141	0.0071	-0.0174	-0.0099	-0.0458	0.0087	-0.0279	-0.0194	0.0151	0.0002
ca	c07	-0.1585	-0.1201	-0.0083	-0.1743	0.0696	-0.2691	-0.2967	-0.0943	-0.1854	-0.0196
h5	h15	0.1958	0.1484	0.1058	0.1561	0.1283	0.2106	0.1868	0.1796	0.1689	0.1477
ca	c08	-0.1585	-0.1201	-0.0083	-0.1743	0.0696	-0.2691	-0.2967	-0.0943	-0.1854	-0.0196
h5	h16	0.1958	0.1484	0.1058	0.1561	0.1283	0.2106	0.1868	0.1796	0.1689	0.1477
c3	c09	-0.2692	-0.0686	0.0418	0.1031	0.0414	-0.0985	-0.0488	0.2551	-0.0228	0.1243
h2	h17	0.1242	0.0650	0.0191	0.0301	0.0240	0.0853	0.0594	-0.0045	0.0394	0.0297
h2	h18	0.1242	0.0650	0.0191	0.0301	0.0240	0.0853	0.0594	-0.0045	0.0394	0.0297
h2	h19	0.1242	0.0650	0.0191	0.0301	0.0240	0.0853	0.0594	-0.0045	0.0394	0.0297
nh	n10	-0.2330	-0.0209	-0.1891	0.1117	-0.1517	-0.2035	0.0242	-0.0349	0.2608	0.0752
ca	c11	0.0114	-0.0387	0.1069	-0.1125	0.1774	-0.1034	-0.1693	-0.1678	-0.2491	-0.0538
ca	c12	0.1259	0.0405	-0.0647	0.0105	-0.1880	0.0770	0.1493	0.0298	0.0881	0.0405
no	n13	0.5064	0.6246	0.6570	0.6589	0.6569	0.6720	0.6747	0.5736	0.6164	0.5561
o	o14	-0.3577	-0.3845	-0.3865	-0.3892	-0.3985	-0.4106	-0.4277	-0.3861	-0.3969	-0.3885
o	o15	-0.3577	-0.3845	-0.3865	-0.3892	-0.3985	-0.4106	-0.4277	-0.3861	-0.3969	-0.3885
ca	c16	-0.1899	-0.0912	-0.2671	0.0248	-0.2053	0.0610	0.0176	-0.0398	-0.0211	-0.2728
s6	s17	0.9988	0.8952	1.0761	0.8017	0.8902	0.9137	1.0459	0.7753	0.9888	0.9550
o	o18	-0.5311	-0.5084	-0.5577	-0.5293	-0.5389	-0.5490	-0.5844	-0.5546	-0.5623	-0.5635
o	o19	-0.5311	-0.5084	-0.5577	-0.5293	-0.5389	-0.5490	-0.5844	-0.5546	-0.5623	-0.5635
ca	c20	0.1259	0.0405	-0.0647	0.0105	-0.1880	0.0770	0.1493	0.0298	0.0881	0.0405
no	n21	0.5064	0.6246	0.6570	0.6589	0.6569	0.6720	0.6747	0.5736	0.6164	0.5561
o	o22	-0.3577	-0.3845	-0.3865	-0.3892	-0.3985	-0.4106	-0.4277	-0.3861	-0.3969	-0.3885
o	o23	-0.3577	-0.3845	-0.3865	-0.3892	-0.3985	-0.4106	-0.4277	-0.3861	-0.3969	-0.3885

Table S25: **ABCG2** atomic charges (e) of the 10 frames extracted from the QM/MM MD simulation of nitralin. For atom labels, see Figure S7.

A.t.	A.l.	1	2	3	4	5	6	7	8	9	10
c3	c01	-0.1001	-0.0956	-0.0956	-0.0956	-0.0956	-0.1011	-0.1011	-0.0956	-0.1001	-0.1001
hc	h01	0.0430	0.0392	0.0392	0.0392	0.0392	0.0430	0.0430	0.0392	0.0430	0.0430
hc	h02	0.0430	0.0392	0.0392	0.0392	0.0392	0.0430	0.0430	0.0392	0.0430	0.0430
hc	h03	0.0430	0.0392	0.0392	0.0392	0.0392	0.0430	0.0430	0.0392	0.0430	0.0430
c3	c02	-0.0954	-0.0834	-0.0834	-0.0834	-0.0834	-0.0934	-0.0934	-0.0834	-0.0954	-0.0954
hc	h04	0.0552	0.0527	0.0527	0.0527	0.0527	0.0545	0.0545	0.0527	0.0555	0.0555
hc	h05	0.0552	0.0527	0.0527	0.0527	0.0527	0.0545	0.0545	0.0527	0.0555	0.0555
c3	c03	0.2113	0.1928	0.1928	0.1928	0.1928	0.2073	0.2073	0.1928	0.2113	0.2113
h1	h06	0.0625	0.0560	0.0560	0.0560	0.0560	0.0580	0.0580	0.0560	0.0625	0.0625
h1	h07	0.0625	0.0560	0.0560	0.0560	0.0560	0.0580	0.0580	0.0560	0.0625	0.0625
c3	c04	0.2113	0.1928	0.1928	0.1928	0.1928	0.2073	0.2073	0.1928	0.2113	0.2113
h1	h08	0.0625	0.0560	0.0560	0.0560	0.0560	0.0580	0.0580	0.0560	0.0625	0.0625
h1	h09	0.0625	0.0560	0.0560	0.0560	0.0560	0.0580	0.0580	0.0560	0.0625	0.0625
c3	c05	-0.0954	-0.0834	-0.0834	-0.0834	-0.0834	-0.0934	-0.0934	-0.0834	-0.0954	-0.0954
hc	h10	0.0552	0.0527	0.0527	0.0527	0.0527	0.0545	0.0545	0.0527	0.0555	0.0555
hc	h11	0.0552	0.0527	0.0527	0.0527	0.0527	0.0545	0.0545	0.0527	0.0555	0.0555
c3	c06	-0.1001	-0.0956	-0.0956	-0.0956	-0.0956	-0.1011	-0.1011	-0.0956	-0.1001	-0.1001
hc	h12	0.0430	0.0392	0.0392	0.0392	0.0392	0.0430	0.0430	0.0392	0.0430	0.0430
hc	h13	0.0430	0.0392	0.0392	0.0392	0.0392	0.0430	0.0430	0.0392	0.0430	0.0430
hc	h14	0.0430	0.0392	0.0392	0.0392	0.0392	0.0430	0.0430	0.0392	0.0430	0.0430
ca	c07	0.1055	0.0785	0.0785	0.0785	0.0785	0.0895	0.0890	0.0785	0.1055	0.1055
h5	h15	0.1755	0.1745	0.1745	0.1745	0.1745	0.1730	0.1730	0.1745	0.1755	0.1755
ca	c08	0.1055	0.0785	0.0785	0.0785	0.0785	0.0895	0.0890	0.0785	0.1055	0.1055
h5	h16	0.1755	0.1745	0.1745	0.1745	0.1745	0.1730	0.1730	0.1745	0.1755	0.1755
c3	c09	-0.4801	-0.4841	-0.4841	-0.4841	-0.4841	-0.4821	-0.4821	-0.4841	-0.4801	-0.4801
h2	h17	0.1204	0.1234	0.1234	0.1234	0.1234	0.1217	0.1217	0.1234	0.1204	0.1204
h2	h18	0.1204	0.1234	0.1234	0.1234	0.1234	0.1217	0.1217	0.1234	0.1204	0.1204
h2	h19	0.1204	0.1234	0.1234	0.1234	0.1234	0.1217	0.1217	0.1234	0.1204	0.1204
nh	n10	-0.8134	-0.8244	-0.8244	-0.8244	-0.8244	-0.8304	-0.8304	-0.8244	-0.8134	-0.8134
ca	c11	0.4950	0.4490	0.4490	0.4490	0.4490	0.4740	0.4740	0.4490	0.4950	0.4950
ca	c12	-0.2657	-0.2202	-0.2202	-0.2202	-0.2202	-0.2397	-0.2397	-0.2202	-0.2657	-0.2657
no	n13	0.6977	0.7027	0.7027	0.7027	0.7027	0.6987	0.6987	0.7027	0.6977	0.6977
o	o14	-0.3853	-0.3783	-0.3783	-0.3783	-0.3783	-0.3795	-0.3795	-0.3783	-0.3853	-0.3853
o	o15	-0.3853	-0.3783	-0.3783	-0.3783	-0.3783	-0.3795	-0.3795	-0.3783	-0.3853	-0.3853
ca	c16	-0.4285	-0.3875	-0.3875	-0.3875	-0.3875	-0.4065	-0.4065	-0.3875	-0.4285	-0.4285
s6	s17	1.3745	1.3695	1.3695	1.3695	1.3695	1.3725	1.3725	1.3695	1.3745	1.3745
o	o18	-0.5765	-0.5730	-0.5730	-0.5730	-0.5730	-0.5750	-0.5750	-0.5730	-0.5765	-0.5765
o	o19	-0.5765	-0.5730	-0.5730	-0.5730	-0.5730	-0.5750	-0.5750	-0.5730	-0.5765	-0.5765
ca	c20	-0.2657	-0.2202	-0.2202	-0.2202	-0.2202	-0.2397	-0.2397	-0.2202	-0.2657	-0.2657
no	n21	0.6977	0.7027	0.7027	0.7027	0.7027	0.6987	0.6987	0.7027	0.6977	0.6977
o	o22	-0.3853	-0.3783	-0.3783	-0.3783	-0.3783	-0.3795	-0.3795	-0.3783	-0.3853	-0.3853
o	o23	-0.3853	-0.3783	-0.3783	-0.3783	-0.3783	-0.3795	-0.3795	-0.3783	-0.3853	-0.3853

Table S26: **RESP-QM/MM** atomic charges (e) of the 10 frames extracted from the QM/MM MD simulation of terbacil. For atom labels, see Figure S8.

A.t.	A.l.	1	2	3	4	5	6	7	8	9	10
c3	c01	-0.0411	0.1334	0.0950	0.0687	-0.0270	0.0580	-0.0555	0.0552	0.0409	0.0493
h1	h01	0.0548	0.0301	0.0303	0.0403	0.0567	0.0337	0.0681	0.0336	0.0379	0.0326
h1	h02	0.0548	0.0301	0.0303	0.0403	0.0567	0.0337	0.0681	0.0336	0.0379	0.0326
h1	h03	0.0548	0.0301	0.0303	0.0403	0.0567	0.0337	0.0681	0.0336	0.0379	0.0326
n	n02	-0.3710	-0.1182	-0.4025	-0.2285	-0.4233	-0.3419	-0.4659	-0.3822	-0.4117	-0.4042
hn	h04	0.3674	0.2827	0.3884	0.3229	0.3510	0.3476	0.3726	0.3671	0.3417	0.3655
c3	c03	0.1349	0.0771	0.1610	0.1219	-0.0102	-0.0716	0.0346	-0.0051	0.0026	0.0920
hc	h05	-0.0633	-0.0590	-0.0773	-0.0737	-0.0339	-0.0181	-0.0363	-0.0550	-0.0476	-0.0477
hc	h06	-0.0633	-0.0590	-0.0773	-0.0737	-0.0339	-0.0181	-0.0363	-0.0550	-0.0476	-0.0477
hc	h07	-0.0633	-0.0590	-0.0773	-0.0737	-0.0339	-0.0181	-0.0363	-0.0550	-0.0476	-0.0477
c3	c04	0.1349	0.0771	0.1610	0.1219	-0.0102	-0.0716	0.0346	-0.0051	0.0026	0.0920
hc	h08	-0.0633	-0.0590	-0.0773	-0.0737	-0.0339	-0.0181	-0.0363	-0.0550	-0.0476	-0.0477
hc	h09	-0.0633	-0.0590	-0.0773	-0.0737	-0.0339	-0.0181	-0.0363	-0.0550	-0.0476	-0.0477
hc	h10	-0.0633	-0.0590	-0.0773	-0.0737	-0.0339	-0.0181	-0.0363	-0.0550	-0.0476	-0.0477
c3	c05	0.1349	0.0771	0.1610	0.1219	-0.0102	-0.0716	0.0346	-0.0051	0.0026	0.0920
hc	h11	-0.0633	-0.0590	-0.0773	-0.0737	-0.0339	-0.0181	-0.0363	-0.0550	-0.0476	-0.0477
hc	h12	-0.0633	-0.0590	-0.0773	-0.0737	-0.0339	-0.0181	-0.0363	-0.0550	-0.0476	-0.0477
hc	h13	-0.0633	-0.0590	-0.0773	-0.0737	-0.0339	-0.0181	-0.0363	-0.0550	-0.0476	-0.0477
cc	c06	0.2623	-0.0628	0.2548	0.0443	0.2337	0.2003	0.2206	0.1970	0.2181	0.2009
cc	c07	-0.2230	0.0744	-0.2604	-0.1771	-0.3169	-0.2236	-0.1898	-0.2502	-0.2149	-0.2295
cl	cl01	-0.1028	-0.1192	-0.1115	-0.0095	-0.0724	-0.0477	-0.1054	-0.0989	-0.0507	-0.0870
c	c08	0.5351	0.2758	0.4963	0.6021	0.5969	0.4197	0.5752	0.6249	0.5925	0.5118
o	o09	-0.5254	-0.5533	-0.5673	-0.5534	-0.5249	-0.5406	-0.5599	-0.5427	-0.5822	-0.5704
n	n10	0.3674	0.2827	0.3884	0.3229	0.3510	0.3476	0.3726	0.3671	0.3417	0.3655
c	c11	0.4929	0.2803	0.3310	0.4413	0.5751	0.4873	0.6144	0.5081	0.5365	0.3829
o	o12	-0.4837	-0.4589	-0.4072	-0.5820	-0.4968	-0.5038	-0.4984	-0.4329	-0.5207	-0.4034
c3	c13	0.5333	0.6309	0.6049	0.6887	0.6637	0.6743	0.5402	0.9875	0.7500	0.2802

Table S27: **ABCG2** atomic charges (e) of the 10 frames extracted from the QM/MM MD simulation of terbacil. For atom labels, see Figure S8.

A.t.	A.l.	1	2	3	4	5	6	7	8	9	10
c3	c01	-0.0779	-0.0769	-0.0779	-0.0769	-0.0779	-0.0779	-0.0769	-0.0769	-0.0779	-0.0779
h1	h01	0.0704	0.0704	0.0704	0.0704	0.0704	0.0704	0.0704	0.0704	0.0704	0.0704
h1	h02	0.0704	0.0704	0.0704	0.0704	0.0704	0.0704	0.0704	0.0704	0.0704	0.0704
h1	h03	0.0704	0.0704	0.0704	0.0704	0.0704	0.0704	0.0704	0.0704	0.0704	0.0704
n	n02	-0.5176	-0.5156	-0.5176	-0.5156	-0.5176	-0.5176	-0.5156	-0.5156	-0.5176	-0.5176
hn	h04	0.4670	0.4670	0.4670	0.4670	0.4670	0.4670	0.4670	0.4670	0.4670	0.4670
c3	c03	-0.1068	-0.1074	-0.1068	-0.1074	-0.1068	-0.1068	-0.1074	-0.1074	-0.1068	-0.1068
hc	h05	0.0520	0.0518	0.0520	0.0518	0.0519	0.0520	0.0518	0.0518	0.0519	0.0520
hc	h06	0.0520	0.0518	0.0520	0.0518	0.0519	0.0520	0.0518	0.0518	0.0519	0.0520
hc	h07	0.0520	0.0518	0.0520	0.0518	0.0519	0.0520	0.0518	0.0518	0.0519	0.0520
c3	c04	-0.1068	-0.1074	-0.1068	-0.1074	-0.1068	-0.1068	-0.1074	-0.1074	-0.1068	-0.1068
hc	h08	0.0520	0.0518	0.0520	0.0518	0.0519	0.0520	0.0518	0.0518	0.0519	0.0520
hc	h09	0.0520	0.0518	0.0520	0.0518	0.0519	0.0520	0.0518	0.0518	0.0519	0.0520
hc	h10	0.0520	0.0518	0.0520	0.0518	0.0519	0.0520	0.0518	0.0518	0.0519	0.0520
c3	c05	-0.1068	-0.1074	-0.1068	-0.1074	-0.1068	-0.1068	-0.1074	-0.1074	-0.1068	-0.1068
hc	h11	0.0520	0.0518	0.0520	0.0518	0.0519	0.0520	0.0518	0.0518	0.0519	0.0520
hc	h12	0.0520	0.0518	0.0520	0.0518	0.0519	0.0520	0.0518	0.0518	0.0519	0.0520
hc	h13	0.0520	0.0518	0.0520	0.0518	0.0519	0.0520	0.0518	0.0518	0.0519	0.0520
cc	c06	0.1094	0.1064	0.1094	0.1064	0.1094	0.1094	0.1064	0.1064	0.1094	0.1094
cc	c07	-0.1202	-0.1182	-0.1202	-0.1182	-0.1202	-0.1202	-0.1182	-0.1182	-0.1202	-0.1202
cl	cl01	-0.1200	-0.1190	-0.1200	-0.1190	-0.1200	-0.1200	-0.1190	-0.1190	-0.1200	-0.1200
c	c08	0.5852	0.5882	0.5852	0.5882	0.5852	0.5852	0.5882	0.5882	0.5852	0.5852
o	o09	-0.5190	-0.5130	-0.5190	-0.5130	-0.5190	-0.5190	-0.5130	-0.5130	-0.5190	-0.5190
n	n10	-0.3414	-0.3434	-0.3414	-0.3434	-0.3414	-0.3414	-0.3434	-0.3434	-0.3414	-0.3414
c	c11	0.6040	0.6010	0.6040	0.6010	0.6040	0.6040	0.6010	0.6010	0.6040	0.6040
o	o12	-0.5640	-0.5670	-0.5640	-0.5670	-0.5640	-0.5640	-0.5670	-0.5670	-0.5640	-0.5640
c3	c13	0.1364	0.1364	0.1364	0.1364	0.1364	0.1364	0.1364	0.1364	0.1364	0.1364

Table S28: **RESP-QM/MM** atomic charges (e) of the 10 frames extracted from the QM/MM MD simulation of imidazole. For atoms labels see Figure S9.

A.t.	A.l.	1	2	3	4	5	6	7	8	9	10
ca	c01	-0.2204	-0.2174	-0.2089	-0.2238	-0.2508	-0.2525	-0.2742	-0.3166	-0.2129	-0.2858
ha	h01	0.1865	0.1830	0.1490	0.1644	0.1881	0.1788	0.1791	0.1946	0.1668	0.1948
ca	c02	0.1596	0.1603	0.1093	0.1821	0.1534	0.2476	0.2511	0.2105	0.1371	0.2048
ha	h02	0.0529	0.0567	0.0859	0.0489	0.0491	0.0507	0.0329	0.0604	0.0758	0.0485
ca	c03	0.2539	0.2436	0.2111	0.2119	0.2412	0.2152	0.2926	0.2409	0.3237	0.2645
h4	h03	0.0745	0.0727	0.0951	0.0946	0.0680	0.0668	0.0630	0.0598	0.0486	0.0630
nb	n04	-0.1939	-0.2087	-0.1743	-0.1191	-0.1906	-0.1447	-0.2525	-0.1386	-0.1815	-0.1894
hn	h04	0.3170	0.3199	0.3274	0.3310	0.3619	0.3406	0.3676	0.3290	0.3171	0.3080
nb	n05	-0.6301	-0.6101	-0.5946	-0.6900	-0.6201	-0.7024	-0.6597	-0.6399	-0.6746	-0.6086

Table S29: **ABCG2** atomic charges (e) of the 10 frames extracted from the QM/MM MD simulation of imidazole. For atom labels, see Figure S9.

A.t.	A.l.	1	2	3	4	5	6	7	8	9	10
ca	c01	-0.2290	-0.2290	-0.2290	-0.2290	-0.2290	-0.2290	-0.2290	-0.2290	-0.2290	-0.2290
ha	h01	0.1580	0.1580	0.1580	0.1580	0.1580	0.1580	0.1580	0.1580	0.1580	0.1580
ca	c02	0.2259	0.2259	0.2259	0.2259	0.2259	0.2259	0.2259	0.2259	0.2259	0.2259
ha	h02	0.0421	0.0421	0.0421	0.0421	0.0421	0.0421	0.0421	0.0421	0.0421	0.0421
ca	c03	0.3824	0.3824	0.3824	0.3824	0.3824	0.3824	0.3824	0.3824	0.3824	0.3824
h4	h03	0.0601	0.0601	0.0601	0.0601	0.0601	0.0601	0.0601	0.0601	0.0601	0.0601
nb	n04	-0.3215	-0.3215	-0.3215	-0.3215	-0.3215	-0.3215	-0.3215	-0.3215	-0.3215	-0.3215
hn	h04	0.3500	0.3500	0.3500	0.3500	0.3500	0.3500	0.3500	0.3500	0.3500	0.3500
nb	n05	-0.6670	-0.6670	-0.6670	-0.6670	-0.6670	-0.6670	-0.6670	-0.6670	-0.6670	-0.6670

Table S30: **RESP-QM/MM** atomic charges (e) of the 10 frames extracted from the QM/MM MD simulation of thiophene. For atom labels, see Figure S10.

A.t.	A.l.	1	2	3	4	5	6	7	8	9	10
cd	c01	-0.1600	-0.1188	-0.1324	-0.1377	-0.1079	-0.1299	-0.0986	-0.1189	-0.1635	-0.1194
hc	h01	0.1143	0.1169	0.1084	0.1097	0.0967	0.1195	0.1127	0.1150	0.1250	0.1039
cc	c02	-0.1600	-0.1188	-0.1324	-0.1377	-0.1079	-0.1299	-0.0986	-0.1189	-0.1635	-0.1194
hc	h02	0.1143	0.1169	0.1084	0.1097	0.0967	0.1195	0.1127	0.1150	0.1250	0.1039
cd	c03	-0.1051	-0.1348	-0.1449	-0.1455	-0.1367	-0.1601	-0.1859	-0.1429	-0.1049	-0.1377
hc	h03	0.1350	0.1481	0.1622	0.1627	0.1408	0.1521	0.1479	0.1530	0.1431	0.1438
cc	c04	-0.1051	-0.1348	-0.1449	-0.1455	-0.1367	-0.1601	-0.1859	-0.1429	-0.1049	-0.1377
hc	h04	0.1350	0.1481	0.1622	0.1627	0.1408	0.1521	0.1479	0.1530	0.1431	0.1438
ss	s05	0.0314	-0.0228	0.0134	0.0216	0.0142	0.0368	0.0477	-0.0122	0.0008	0.0188

Table S31: **ABCG2** atomic charges (e) of the 10 frames extracted from the QM/MM MD simulation of thiophene. For atom labels, see Figure S10.

A.t.	A.l.	1	2	3	4	5	6	7	8	9	10
cd	c01	-0.1390	-0.1390	-0.1390	-0.1390	-0.1390	-0.1390	-0.1390	-0.1390	-0.1390	-0.1390
hc	h01	0.1340	0.1340	0.1340	0.1340	0.1340	0.1340	0.1340	0.1340	0.1340	0.1340
cc	c02	-0.1390	-0.1390	-0.1390	-0.1390	-0.1390	-0.1390	-0.1390	-0.1390	-0.1390	-0.1390
hc	h02	0.1340	0.1340	0.1340	0.1340	0.1340	0.1340	0.1340	0.1340	0.1340	0.1340
cd	c03	-0.2960	-0.2960	-0.2960	-0.2960	-0.2960	-0.2960	-0.2960	-0.2960	-0.2960	-0.2960
hc	h03	0.1510	0.1510	0.1510	0.1510	0.1510	0.1510	0.1510	0.1510	0.1510	0.1510
cc	c04	-0.2960	-0.2960	-0.2960	-0.2960	-0.2960	-0.2960	-0.2960	-0.2960	-0.2960	-0.2960
hc	h04	0.1510	0.1510	0.1510	0.1510	0.1510	0.1510	0.1510	0.1510	0.1510	0.1510
ss	s05	0.2980	0.2980	0.2980	0.2980	0.2980	0.2980	0.2980	0.2980	0.2980	0.2980

Table S32: **RESP-QM/MM** atomic charges (e) of the 10 frames extracted from the QM/MM MD simulation of 3-methyl-pyridine. For atom labels, see Figure S11.

A.t.	A.l.	1	2	3	4	5	6	7	8	9	10
c3	c01	0.0941	0.0667	0.0944	-0.0399	-0.2348	0.1504	0.2021	0.1936	0.1999	0.0443
hc	h01	0.0059	0.0209	-0.0028	0.0360	0.0905	0.0008	-0.0167	-0.0110	-0.0235	0.0002
hc	h02	0.0059	0.0209	-0.0028	0.0360	0.0905	0.0008	-0.0167	-0.0110	-0.0235	0.0002
hc	h03	0.0059	0.0209	-0.0028	0.0360	0.0905	0.0008	-0.0167	-0.0110	-0.0235	0.0002
ca	c02	0.3428	0.4488	0.2003	0.3040	0.2236	0.3001	0.4085	0.3252	0.3267	0.2856
h4	h04	0.0043	0.0105	0.0534	0.0370	0.0444	0.0229	-0.0339	0.0020	0.0137	0.0208
ca	c03	0.1994	0.2906	0.2777	0.3782	0.4481	0.3666	0.3596	0.3429	0.4279	0.3388
h4	h05	0.0505	0.0117	0.0494	-0.0131	-0.0487	-0.0102	0.0033	0.0169	0.0071	0.0190
ca	c04	-0.1573	-0.2896	-0.3344	-0.3319	-0.3538	-0.2582	-0.2727	-0.2904	-0.4253	-0.3002
ha	h06	0.1034	0.1303	0.1298	0.1738	0.1516	0.1450	0.1180	0.1104	0.1633	0.1609
ca	c05	-0.0996	0.0592	-0.0460	0.0373	0.0331	0.0680	-0.0334	0.0229	0.0410	-0.0159
ha	h07	0.1281	0.1133	0.1438	0.1279	0.1046	0.0777	0.1255	0.1147	0.0898	0.1250
ca	c06	-0.0746	-0.2080	-0.0178	-0.0889	0.0043	-0.1268	-0.1507	-0.1751	-0.0985	-0.0341
nb	n07	-0.6088	-0.6963	-0.5419	-0.6923	-0.6436	-0.7380	-0.6761	-0.6301	-0.6750	-0.6449

Table S33: **ABCG2** atomic charges (e) of the 10 frames extracted from the QM/MM MD simulation of 3-methyl-pyridine. For atom labels, see Figure S11.

A.t.	A.l.	1	2	3	4	5	6	7	8	9	10
c3	c01	-0.0508	-0.0508	-0.0508	-0.0508	-0.0508	-0.0508	-0.0508	-0.0508	-0.0508	-0.0508
hc	h01	0.0470	0.0470	0.0470	0.0470	0.0470	0.0470	0.0470	0.0470	0.0470	0.0470
hc	h02	0.0470	0.0470	0.0470	0.0470	0.0470	0.0470	0.0470	0.0470	0.0470	0.0470
hc	h03	0.0470	0.0470	0.0470	0.0470	0.0470	0.0470	0.0470	0.0470	0.0470	0.0470
ca	c02	0.3259	0.3259	0.3269	0.3269	0.3269	0.3269	0.3269	0.3269	0.3269	0.3269
h4	h04	0.0211	0.0211	0.0211	0.0211	0.0211	0.0211	0.0211	0.0211	0.0211	0.0211
ca	c03	0.3209	0.3209	0.3219	0.3219	0.3219	0.3219	0.3219	0.3219	0.3219	0.3219
h4	h05	0.0211	0.0211	0.0211	0.0211	0.0211	0.0211	0.0211	0.0211	0.0211	0.0211
ca	c04	-0.1590	-0.1590	-0.1590	-0.1590	-0.1590	-0.1590	-0.1590	-0.1590	-0.1590	-0.1590
ha	h06	0.1240	0.1240	0.1240	0.1240	0.1240	0.1240	0.1240	0.1240	0.1240	0.1240
ca	c05	-0.0770	-0.0770	-0.0780	-0.0780	-0.0780	-0.0780	-0.0780	-0.0780	-0.0780	-0.0780
ha	h07	0.1190	0.1190	0.1190	0.1190	0.1190	0.1190	0.1190	0.1190	0.1190	0.1190
ca	c06	-0.1263	-0.1263	-0.1263	-0.1263	-0.1263	-0.1263	-0.1263	-0.1263	-0.1263	-0.1263
nb	n07	-0.6610	-0.6610	-0.6620	-0.6620	-0.6620	-0.6620	-0.6620	-0.6620	-0.6620	-0.6620
